# Monolayer Amorphous Carbon: Unlocking Disorder‐Induced Lithiophilicity

**DOI:** 10.1002/advs.202516490

**Published:** 2025-11-25

**Authors:** Lu Shi, Hanning Zhang, Artem K. Grebenko, Ruslan Yamaletdinov, Rejaul SK, Ranjith Shivajirao, Zheng Jue Tong, Sergey Luchkin, Hongji Zhang, Konstantin V. Iakoubovskii, Alena A. Alekseeva, Andrei Starkov, Carlo M. Orofeo, Junhao Lin, Kazutomo Suenaga, Chee‐Tat Toh, Remi Mahfouz, Talah M. Tayeb, Nada Qari, Stefan Adams, Bent Weber, Oleg V. Yazyev, Barbaros Özyilmaz

**Affiliations:** ^1^ Department of Materials Science and Engineering National University of Singapore Singapore 117575 Singapore; ^2^ Department of Physics National University of Singapore Singapore 117551 Singapore; ^3^ Institute of Physics Ecole Polytechnique Fédérale de Lausanne (EPFL) Lausanne CH‐1015 Switzerland; ^4^ Division of Physics and Applied Physics School of Physical and Mathematical Sciences Nanyang Technological University Singapore 637371 Singapore; ^5^ Skolkovo Institute of Science and Technology Bolshoy Boulevard 30, bldg. 1 Moscow 121205 Russia; ^6^ Institute for Functional Intelligent Materials National University of Singapore Singapore 117544 Singapore; ^7^ Department of Physics State Key Laboratory of Quantum Functional Materials and Guangdong Basic Research Center of Excellence for Quantum Science Southern University of Science and Technology (SUSTech) Shenzhen 518055 China; ^8^ Sanken Osaka University Ibaraki 567‐0047 Japan; ^9^ Centre for Advanced 2D Material Singapore National University of Singapore Singapore 117546 Singapore; ^10^ Research & Development Center Saudi Aramco, Dhahran 31311 Saudi Arabia

**Keywords:** amorphous carbon, anode‐less batteries, lithiophilic coatings, lithium nucleation, structural disorder

## Abstract

Dendritic lithium growth on the current collector remains a major obstacle to developing anode‐less batteries, arising from inhomogeneous lithium nucleation and uneven surface lithiophilicity. Existing approaches, such as metallic or carbonaceous interlayers, often fail to stabilize lithium deposition due to mechanical degradation or spatial variations in lithium affinity. Here, we demonstrate that a monolayer amorphous carbon (MAC) film—a single‐atom‐thick disordered sp^2^ network grown directly on copper—can fundamentally alter lithium nucleation behavior. The topological disorder of MAC produces a dense distribution of electron‐rich sites that uniformly strengthen lithium binding. As a result, the MAC surface exhibits a lithium contact angle of 31 ± 5°, four times lower than that of graphene and nearly three times lower than that of bare copper, leading to homogeneous wetting and deposition. Electrochemical tests reveal a reduced nucleation overpotential of 28.9 mV at 0.5 mA cm^−2^. Density functional theory and scanning tunneling microscopy confirm that disorder‐induced localization of states near the Fermi level enhances electronegativity and forms continuous lithium‐binding sites. These findings establish intrinsic structural disorder, rather than chemical doping, as an effective route to designing uniformly lithiophilic current collectors for next‐generation anode‐less batteries.

## Graphical abstract

Atomic‐scale disorder in monolayer amorphous carbon generates abundant Li binding sites. Combined contact angle, electrochemical, and Density Functional Theory analyses show how this broad distribution of binding energies lowers the contact angle to around 30°, reduces the nucleation overpotential, and yields uniform lithium deposition. The study establishes disorder‐tuned two‐dimensional amorphous carbon as a route to controlled metal nucleation.

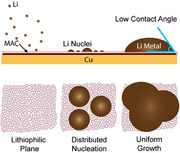

## 1 Introduction

Rising global energy demand is driving battery research toward high‐energy chemistries, most notably anode‐less lithium‐metal batteries. These cells deliver higher energy density and improved safety by plating lithium (Li) directly onto the current collector rather than using a thick Li foil.^[^ ^1^, ^2^ ^]^ However, Li deposition on copper (Cu), a traditionally used current collector material, is hindered by two fundamental issues: dendritic growth and formation of dead Li, both of which cause capacity loss and safety issues.^[^ ^3^ ^]^ Approaches to mitigate dendrites include electrolyte modification,^[^ ^4^, ^5^, ^6^ ^]^ artificial solid electrolyte interphase (SEI) engineering,^[^ ^7^, ^8^, ^9^ ^]^ and, most relevant to this work, lithiophilic surface coatings^[^ ^10^, ^11^ ^]^ that guide uniform Li nucleation. A coating is seen as lithiophilic if it lowers the Li–substrate interfacial free energy and exhibits small molten Li contact angles, low nucleation overpotentials (NOP), uniform large Li deposits, and facilitates the formation of a stable SEI with low ion diffusion resistance.^[^ ^12^, ^13^, ^14^, ^15^ ^]^ These traits initiate Li deposition uniformly and readily, suppressing local current hot spots that trigger dendrite formation.^[^ ^14^, ^16^ ^]^

While Li‐alloyable metallic coatings such as indium, zinc, and silver films can promote uniform initial Li deposition, their long‐term stability is a critical failure point.^[^ ^17^ ^]^ Ultrathin Au or Pt interlayers, for example, rapidly agglomerate into micrometer‐scale Li‐metal alloy clusters that become embedded in the deposits, trapping active Li and lowering Coulombic efficiency (CE).^[^ ^18^, ^19^ ^]^ In addition, these metal coatings often suffer from volume expansion during alloying and dealloying cycles, which induce film cracking and eventual delamination from the current collector, compromising structural integrity.^[^ ^20^, ^21^ ^]^ Thus, despite their strong lithiophilicity, alloy‐based coatings face considerable practical challenges in ensuring stable, dendrite‐free Li deposition.

Carbon offers a chemically stable and abundant alternative,^[^ ^22^, ^23^, ^24^, ^25^, ^26^, ^27^ ^]^ with thin films that can be grown directly on current collectors^[^ ^28^, ^29^, ^30^ ^]^ or serve as current collectors themselves.^[^ ^31^, ^32^, ^33^, ^34^ ^]^ However, conventional carbon‐based approaches still face challenges. For instance, pristine graphitic carbons, including atomically thin graphene, have inherently inert basal planes that weakly bind Li, making them less ideal for anode‐less battery applications. Attempts to enhance Li affinity via heteroatom doping have successfully introduced localized lithiophilic sites,^[^ ^28^, ^29^, ^35^ ^]^ yet typically with limited doping concentrations (generally below 10%).^[^ ^36^, ^37^, ^38^ ^]^ Moreover, due to the chemical inertness of basal C─C bonds, dopants often segregate preferentially at edges and defect sites,^[^ ^39^, ^40^, ^41^, ^42^, ^43^ ^]^ leaving large portions of the basal plane lithiophobic. Most importantly, there is a fundamental scale mismatch: the smallest stable Li nucleus is ≈30–40 nm in diameter,^[^ ^44^, ^45^, ^46^ ^]^ which significantly exceeds the size of isolated atomic dopants,^[^ ^47^ ^]^ making it unclear whether dispersed active sites alone can effectively guide uniform deposition. In contrast, the topological disorder of MAC naturally distributes lithiophilic sites throughout its sp^2^ network. This provides a more continuous landscape for Li binding across tens of nanometers length scales, which helps to mitigate the scale mismatch that limits doped carbons. Additionally, doped carbon materials often exist as powders, requiring binders and additives,^[^ ^28^ ^]^ that introduce extra thickness and inactive mass, counterproductive to the goals of anode‐less batteries. These limitations underscore the need for atomically thin carbon coatings that deliver continuous Li affinity across the relevant length scales.

Here, we introduce MAC as a material that overcomes these challenges by leveraging intrinsic structural disorder rather than extrinsic doping. Grown directly on Cu, this dopant‐free film presents a topologically disordered sp^2^ network that functions as a uniformly lithiophilic surface. Experiment and Density Functional Theory (DFT) calculations show that MAC significantly enhances the Li adhesion energy across the entire surface: 100% of sites bind Li more strongly than the Li(111) surface (−1.75 eV) or graphene (−1.37 eV), with adhesion energies reaching as high as −3.34 eV, a value comparable to the most active sites in doped graphitic carbons.^[^ ^28^, ^48^ ^]^ The key descriptors associated with the change of the Li adhesion energy are the higher values of electronegativity^[^ ^49^ ^]^ and local density of states (LDOS) close to the Fermi level, which we further verified using scanning tunneling microscopy and spectroscopy (STM/STS). This intrinsic, widespread lithiophilicity translates directly to superior electrochemical performance. We demonstrate a fourfold reduction in the molten Li contact angle, a significantly lower NOP, and a threefold extension in half‐cell cycling life compared to bare Cu current collectors. Beyond its electrochemical advantages, MAC shows ultra‐strong adhesion to Cu,^[^ ^49^ ^]^ high fracture toughness,^[^ ^50^ ^]^ and large Young's modulus,^[^ ^51^ ^]^ (Note SI, Supporting Information), ensuring the mechanical robustness required for a promising interfacial layer for current collectors.

## 2 Results and Discussion

**Figure 1:**
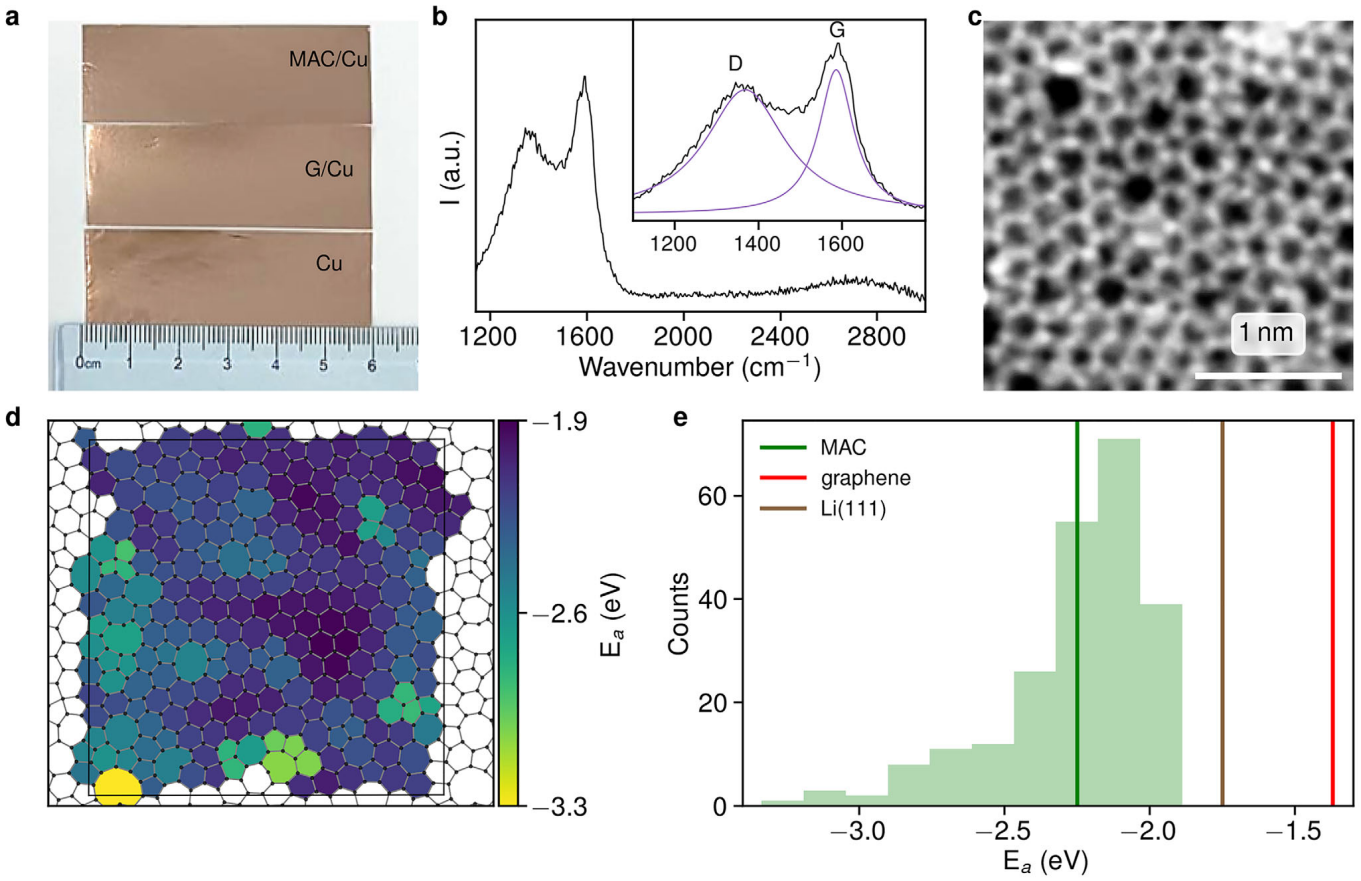
MAC as a carbon‐based current collector coating with high Li affinity. a) Optical images of 2 × 6 cm Cu foils used as substrates: top: Cu foil covered with MAC (MAC/Cu); middle: reference sample of Cu foil covered with graphene (G/Cu); bottom: reference sample of bare Cu foil. b) Raman spectrum of MAC transferred onto SiO_2_, showing a negligible and broadened 2D band. The inset shows fits to D and G bands with Gaussian and Breit‐Wigner‐Fano functions, respectively. I_D_/I_G_ = 0.82, which gives an estimate of 1.2 nm average crystallite size. c) TEM image of freestanding MAC, revealing crystallites containing 2D continuous random carbon network. Note that the larger voids are due to the presence of 8‐member rings and not due to structural defects, which give rise to functional groups. d) Spatial map of Li adhesion energy (E_a_) to various rings in the MAC structure reconstructed from the TEM data. e) Histogram of the distribution of E_a_ values across the lattice, with reference lines denoting values for pristine graphene (red line, −1.37 eV), Li(111) (brown line, −1.75 eV), and the average E_a_ (green line, −2.25 eV). The values for graphene and Li(111) are given for reference.

Pristine Cu foil and Cu foil coated with chemical vapor deposition grown graphene served as control electrodes. The graphene layer was identified and characterized by Raman spectroscopy and transmission electron microscopy (TEM) (Figure S1, Supporting Information). Pristine Cu is moderately lithiophilic,^[^ ^50^ ^]^ whereas graphene is lithiophobic.^[^ ^51^ ^]^ To ensure a clean surface, Cu foils were first washed and annealed in a reducing atmosphere to remove surface oxides (See Experimental Section). MAC/Cu foil, graphene/Cu (G/Cu) foil, and Cu foil (**Figure** **1**a) all exhibited a similar optical appearance. The Raman spectrum of MAC (Figure 1b) showed a broadened G‐band centered at 1580 cm^−1^ and an intense D‐band at 1320 cm^−1^, with an I_D_/I_G_ ratio of ≈0.82. Figure 1b, These spectral features indicate a high degree of structural disorder with crystallite size of approximate 1.2 nm.^[^ ^52^, ^53^, ^54^ ^]^ The detailed atomic structure was analyzed using high‐resolution TEM (Figure 1c). As can be seen from the TEM images, the MAC structure consists of distorted nano‐crystallites within a continuous disordered network of five‐ to eight‐membered rings as established previously.^[^ ^54^, ^55^ ^]^ The MAC–Cu interface exhibits covalent‐like Cu─C bonding, arising from the corrugated morphology of MAC^[^ ^49^ ^]^ (high‐resolution TEM cross‐sectional image shown in Figure S2, Supporting Information). Electronically, MAC is intrinsically in‐plane insulating, with a sheet resistivity on the order of 10^11^ Ω sq^−1^ at room temperature.^[^ ^54^ ^]^ In contrast, two‐terminal out‐of‐plane transport measurements (Figures S3 and S4, Supporting Information) show that its specific out‐of‐plane resistance is 6.53 × 10^−5^ Ω·cm^2^, comparable to that of graphene,^[^ ^56^ ^]^ indicating seamless charge transfer across the MAC–Cu interface.

To understand the unique Li nucleation behavior observed on the disordered MAC surface, we developed a structural model that reproduces the five‐ to eight‐membered ring statistics obtained from TEM analysis (see Figure 1d, Computational Section). A site‐resolved Li adhesion energy map reveals pronounced local variability across the amorphous carbon network. Nano‐crystalline domains composed predominantly of hexagonal rings exhibit the weakest Li affinity, with an average Li adhesion energy of −2.14 eV—still significantly stronger than that of pristine graphene (−1.37 eV; Figure 1e). In contrast, amorphous regions display enhanced lithiophilicity, with pentagonal rings (−2.44 eV) binding Li more strongly than heptagonal ones (−2.25 eV). The most lithiophilic sites are found at specific pentagons, as well as at isolated octagonal and decagonal motifs reaching Li adhesion energies as low as −3.34 eV, comparable in activity to the most reactive sites in doped graphitic carbons.

The large variations in Li adhesion energy can be accurately described by a modified hard‐soft acid‐base (HSAB)^[^ ^57^ ^]^ theory generalized to include electrostatic and screening effects (Note SII, Supporting Information). We express the adhesion energy of a Li atom to the MAC surface as:

(1) $$E_{a}=-\frac{{\left(\mu_{Li}-\mu_{MAC}\right)}^{2}}{2\left(\eta_{Li}+\eta_{MAC}\right)}$$

Here, µ is the chemical potential and η is the hardness. The chemical potentials are defined as $\mu_{Li}=\mu_{Li}^{0}+\sum n_{i}^{0}/d_{i}$ and $\mu_{MAC}=\mu_{MAC}^{0}$, where $\mu_{Li/\mathrm{MAC}}^{0}$ represents the Mulliken electronegativity of each species. The $\sum n_{i}^{0}/d_{i}$ correction term captures site‐dependent shifts in the Li chemical potential due to initial charge inhomogeneities $n_{i}^{0}$ on the bare MAC surface, with *d_i_* denoting the distance from each carbon atom to the Li atom. The modified hardnesses are given by: $\eta_{Li}=1/g_{Li}+\sum \tilde{n_{i}}/d_{i}$, and $\eta_{MAC}=1/g_{MAC}$, where *g*_*Li*/*MAC*_ are the total density of states at the Fermi level. The $\sum \tilde{n_{i}}/d_{i}$ term accounts for the site‐dependent screening response of MAC, with $\tilde{n_{i}}\;$ being the induced charge at carbon atom *i*, due to a unit test charge placed at the adhesion site.

**Figure 2:**
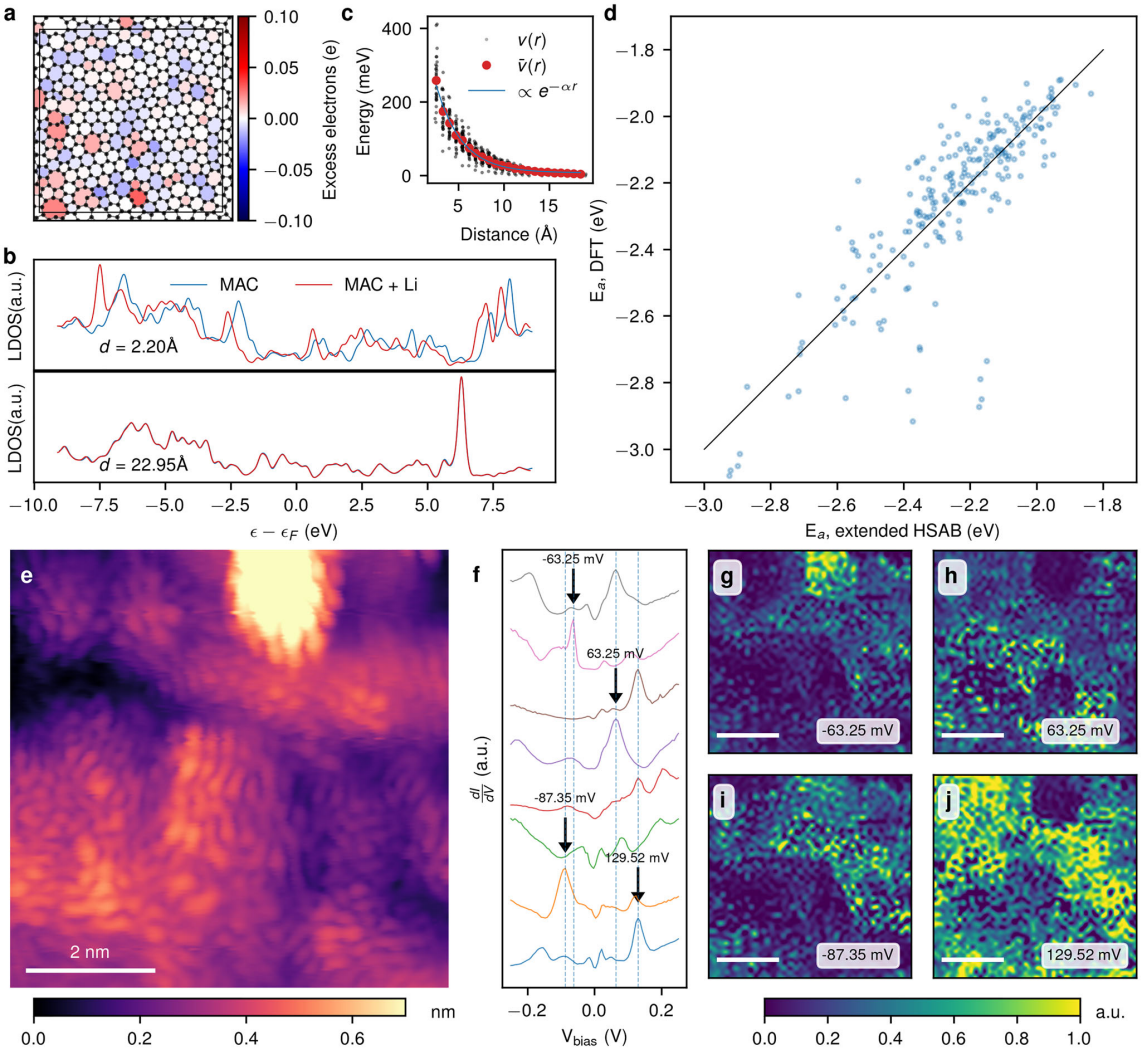
Role of LDOS and electronegativity in the lithiophilicity and STM analysis of LDOS in MAC. a) Spatial variation of initial Mulliken charges per ring (*n* ^0^) across the MAC surface. b) LDOS changes in MAC, depending on the distance to the Li adatom. Atoms in close proximity to Li exhibit noticeable shifts of the spectrum, which decays rapidly and becomes negligible at larger distances. c) LDOS shift (*v*) at various MAC sites plotted against their distance (r) from the adsorbed Li atom. The decay of band shifts follows nearly exponential behavior, indicating strong localization effects. The blue line represents an exponential decay fit. d) Correlation between the adsorption energies calculated by DFT (E_a,_ DFT) and by a modified hard‐soft acid‐base (E_a,_ extended HSAB) model. e) STM topography image of MAC sample measured at V_B_ = 600 mV, I_t_ = 200pA. f) LDOS spectra taken at random points showing rich variability of states in the vicinity of Fermi level. g–j) STS LDOS maps at energy levels indicated (arrows) in panel b for occupied (g, i) and unoccupied (h, j) states. The scale bar is 2 nm on every panel.

Our analysis reveals two key descriptors that govern lithiophilicity. First, local charge affinity: certain ring motifs (particularly pentagons) accumulate excess negative charge (**Figure** **2**a), which increases the Li chemical potential via the $\sum n_{i}^{0}/d_{i}$ term and strengthens Li binding at these adsorption sites. In contrast, motifs such as heptagons are associated with a relative deficit of negative charge, lowering the chemical potential and reducing Li affinity. Interestingly, amorphous regions exhibit a greater local charge surplus compared to crystalline domains on average (Figure S5, Supporting Information), consistent with the enhanced electronegativity^[^ ^49^ ^]^ and lithiophilicity of MAC. MAC's disordered network naturally provides many electron‐excess spots across the film, which enhances its overall ability to attract and stabilize Li.

Second, the LDOS: a high LDOS at the adsorption site enables more efficient electronic screening response, concentrating induced charges $\tilde{n_{i}}$ closer to the Li ion. This leads to a more negative $\sum \tilde{n_{i}}/d_{i}$, reducing the site's hardness and enhancing Li binding. Physically, the interaction with the Li atom induces a local screening potential *v_i_*, which effectively shifts the electronic spectrum (Figure 2b). The induced charge can then be estimated as

(2) $$\tilde{n_{i}}=\;\frac{n_{i}}{\sum n_{i}}=\frac{v_{i}g_{i}}{\sum v_{i}g_{i}}=\frac{{\bar{v}}_{i}g_{i}}{\sum v_{i}g_{i}}$$

The non‐self‐consistent approximation replaces the site‐dependent screening potential *v_i_* by an average ${\bar{v}}_{i}$ computed over 45 different Li adsorption sites (Figure 2c), found to follow an exponentially decaying form ${\bar{v}}_{i}=e^{-\alpha d_{i}}$. Notably, non‐hexagonal rings (particularly octagons) exhibit a strong reduction of the hardness term (Figure S6, Supporting Information).

To validate the model, we computed Li adhesion energies and compared them with DFT results. As shown in Figure 2d, the model accurately captures site‐specific trends governed by local charge inhomogeneity and density of states, confirming its reliability for predicting lithiophilicity in MAC. Rare (< 4%) outliers (discrepancy > 0.3 eV) almost exclusively are due to an underestimation of the adhesion energy caused by partially covalent bond formation (Figures S7 and S8, Supporting Information). So far, the significantly enhanced lithiophilicity of MAC is attributable to abundant atomic‐scale active sites uniformly distributed across its surface through electronegativity and LDOS.

Experimentally, enhanced electronegativity for MAC on the surface of Cu has been reported elsewhere.^[^ ^49^ ^]^ Further, we verified the rich spectral and spatial distribution of LDOS in MAC using STM and STS.

MAC samples for STM experiments were grown on copper foil and then transferred onto flash‐annealed gold on mica substrates. After transfer, samples were annealed in the ultra‐high vacuum chamber of the STM setup to remove atmospheric contamination. This process provided a clean MAC surface on an atomically flat crystalline gold substrate, which enabled reliable atomic‐resolution imaging of the MAC surface (Figure 2e). Using STS, we measured the LDOS at multiple points across the surface by recording dI/dV spectra with a lock‐in amplifier with 10 mV modulation and *f* = 752 Hz (see Figure 2f). These typical spectra show a large enhancement of the LDOS in the vicinity of the Fermi level. To further examine spatial variations, we mapped the LDOS in a grid‐spectroscopy mode over a 7 × 7 nm area with 50 × 50 points. These maps reveal a complex electronic landscape with regions of localized and extended states on the scale of an individual nucleus. We observed this heterogeneous enhancement of the LDOS for occupied (Figure 2g,i) and unoccupied (Figure 2h,j) electronic states, distributed uniformly across the measured area.

The presence of these LDOS‐rich regions across a wide energy range indicates that the MAC surface offers numerous active sites, which is consistent with its high lithiophilicity observed in our electrochemical measurements. In the context of MAC, the STS maps provide the first direct experimental evidence that amorphous‐carbon regions of locally high LDOS are the preferred docking sites for Li—a novel demonstration of the LDOS–lithiophilicity correlation in a disordered two‐dimensional (2D) material.

The HSAB *E_a_* expression aligns with the known results that 2D surfaces with higher DOS near the lowest unoccupied state tend to bind Li more strongly,^[^ ^57^, ^58^ ^]^ and describes the tendency of negatively charged, high‐LDOS dopants in carbon to promote Li nucleation.^[^ ^28^ ^]^

**Figure 3:**
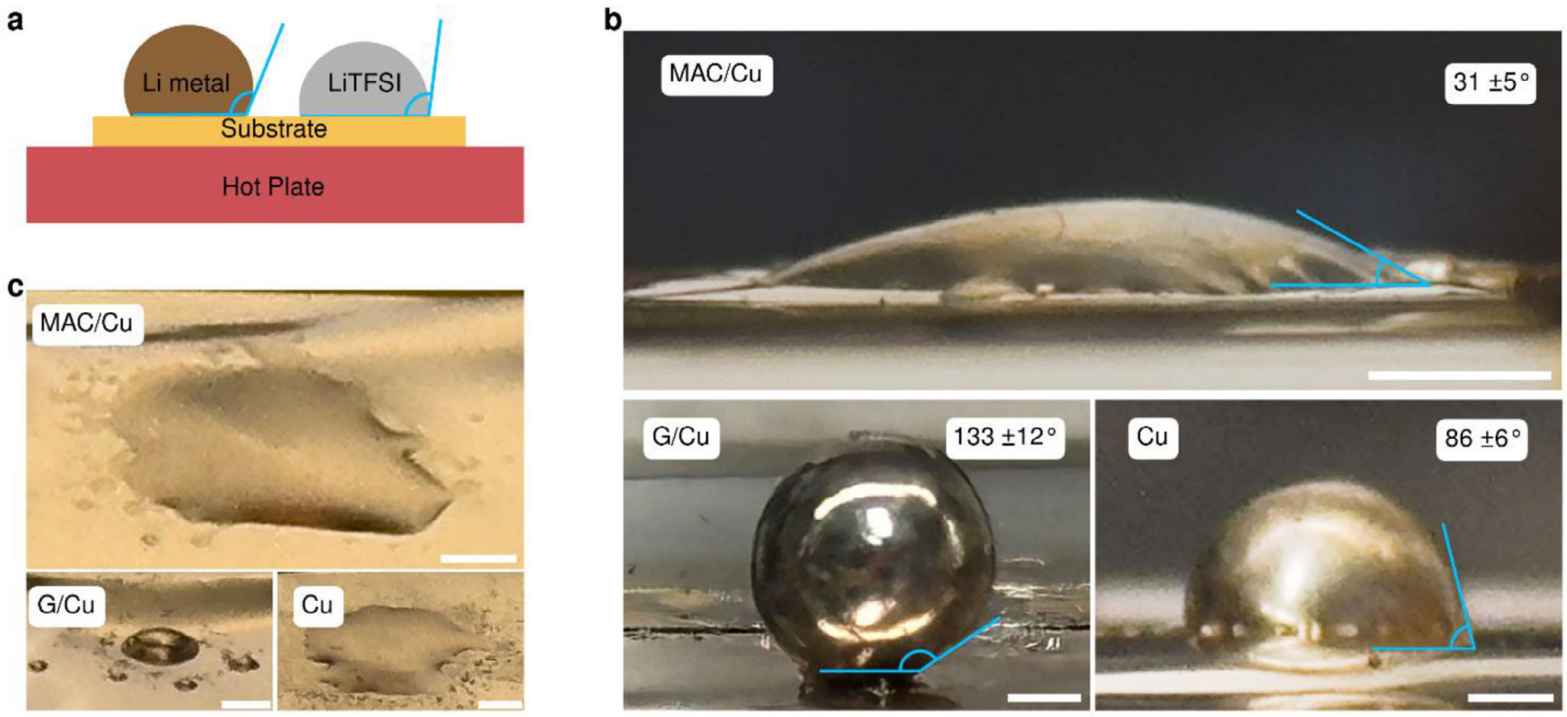
Li^+^/Li contact angle measurements. a) Schematic of the experiment. The Li pellet or LiTFSI powder is placed on a heated substrate (250 °C) to evaluate wettability. b) Optical images of contact angle measurements for molten Li on MAC/Cu, G/Cu, and Cu. Scale bar, 1 mm. c) Optical images of melted LiTFSI on MAC/Cu, G/Cu, and Cu. Scale bar, 2 mm.

Next, we assess MAC's affinity for both metallic Li^[^ ^59^, ^60^, ^61^ ^]^ and a commonly used Li metal battery electrolyte salt, lithium bis(trifluoromethanesulfonyl)imide (LiTFSI), through contact angle measurements. In our study, for Li metal, we folded a piece of Li foil, gently compressed it into a roughly spherical pellet, placed it on each substrate, and heated it to 250 °C (**Figure** **3**a). Optical images of the resulting molten Li droplets were taken for each substrate (Figure 3b; Figure S9, Supporting Information). It is important to note that the Li contact angle depends on temperature.^[^ ^51^ ^]^ However, comparisons between different substrates remain valid under identical experimental conditions. Here, we observed a remarkably low contact angle of 31 ± 5° for MAC on Cu, indicative of strong intrinsic Li‐substrate interaction. In contrast, graphene on Cu, commonly regarded as lithiophobic due to its chemically inert basal plane, had a significantly higher contact angle of 133 ± 12°. Bare Cu, after oxide removal, showed an intermediate contact angle of 86 ± 6°, aligning with recent reports that attribute moderate lithiophilicity to clean Cu surfaces.^[^ ^50^ ^]^ This qualitative contrast highlights the stronger Li affinity of MAC, with the molten Li contact angle serving as a comparative metric of intrinsic substrate–‐Li affinity, correlated with the charge‐transfer resistance discussed further.

The enhanced adhesion of Li on MAC can again be described by the HSAB model. Employing the classical formulation by dropping the site‐dependent electrostatic and screening terms, we take system A as bulk Li and system B as graphene or MAC. We observe a notable increase in the electronegativity difference with Li, from 1.44 eV for graphene to 1.78 eV for MAC, with a simultaneous decrease of the hardness terms through the enhancement in the density of states at the Fermi level. Both factors contribute to a higher HSAB predicted interfacial Li adhesion energy of 55 meV Å^−2^ for MAC, compared to 19 meV Å^−2^ for graphene (Note SIII, Supporting Information). These predictions closely match the DFT‐calculated energies of 64 meV Å^−2^ for MAC and 21 meV Å^−2^ for graphene and correspond to the reduction of the contact angle from 119° for graphene to 58° for MAC. In our calculations, electrochemical interfacial effects were not explicitly considered; however, their influence on the contact angle is expected to be minor—typically within 5°.^[^ ^62^, ^63^, ^64^ ^]^

The affinity for melted LiTFSI (melting point: 234 °C) was also assessed by placing its powder on all three surfaces, followed by heating it up to 250 °C (Figure 3a). Tilted‐top‐down optical images were captured at ≈30° viewing angles to enhance visibility (Figure 3c). We observed that melted LiTFSI spreads readily on MAC/Cu and Cu but remains as a droplet on G/Cu. Accurate contact angle measurements for MAC/Cu and Cu were challenging due to the salt's transparency, the constraints of glovebox operation, and the low apparent angles. Still, we conservatively estimate the contact angle to be <10° for both MAC/Cu and Cu, while that on G/Cu is ≈ 30°. This qualitative contrast highlights the stronger salt affinity of MAC, consistent with its disorder‐induced lithiophilicity. It should be emphasized that in real batteries, LiTFSI is dissolved in a solvent rather than used as pure melted salt. Our test therefore, cannot reproduce electrolyte conditions but provides a comparative indicator of surface affinity for the Li salt.

The significantly reduced contact angle of molten Li and LiTFSI observed on MAC is attributable to abundant atomic‐scale active sites uniformly distributed across its surface. Achieving similarly high lithiophilicity typically requires structural modification or doping in sp^2^‐dominated materials like graphene^[^ ^60^, ^65^ ^]^ or hexagonal boron nitride (h‐BN).^[^ ^59^ ^]^

**Figure 4:**
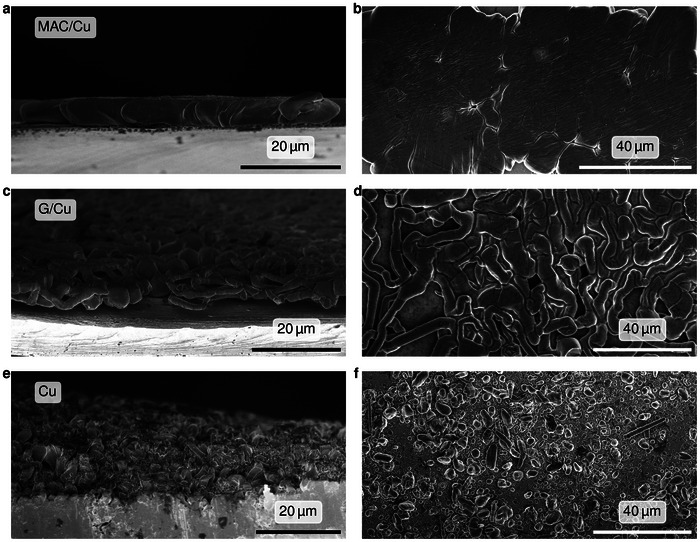
Comparative SEM analysis of Li deposition on MAC/Cu, G/Cu, and Cu foil at 0.5 mA cm^−2^ for 1 h. a,b) MAC/Cu, c,d) G/Cu, and e,f) bare Cu; left images are cross‐sectional, right images are top‐down views. a,b) MAC/Cu: a continuous and flat Li layer composed of low‑aspect‑ratio platelets that coalesce laterally. c,d) G/Cu: Discontinuous, highly nonuniform deposition characterized by sparse, intertwined whiskers. e,f) Cu: denselypacked, high‑aspect‑ratio granular columns that produce a rough and porous film.

We further evaluated the lithiophilicity of each substrate by analyzing the morphology of the deposited Li in coin cells (schematic in Figure S10, Supporting Information). After plating Li for 1 h at 0.5 mA cm^−2^, the Li morphology on MAC/Cu occurred to be markedly different from that on G/Cu or Cu. Cross‑sectional scanning electron microscopy (SEM) analysis (**Figure** **4**a) reveals that Li is deposited on MAC/Cu as a uniform, relatively flat layer. A top‐down view SEM image of the same sample (Figure 4b) reveals large, smooth Li islands with edges that merge into one another, suggesting highly uniform nucleation and lateral growth. Large‐area SEM images of MAC/Cu (Figures S11 and S12, Supporting Information) further confirm the exceptional uniformity of Li nucleation and growth across millimeter‐scale regions. In contrast, G/Cu yields highly nonuniform deposition (Figure 4c,d). Li was deposited as a patchwork of scattered, interconnected whisker networks, with parts of the surface left exposed. This nonuniform coverage agrees with earlier reports that graphene's atomically flat basal plane is intrinsically lithiophobic, and that Li nucleates almost exclusively at grain boundaries and defect sites‐regions of higher chemical reactivity, rather than across the intact lattice.^[^ ^65^ ^]^ Additionally, local delamination of Li whiskers from G/Cu indicates poor adhesion of Li deposits (Figure S13, Supporting Information). On bare Cu (Figure 4e,f), Li grows into densely packed, high‑aspect‑ratio granular columns, creating a structure that is prone to dendritic protrusion.

**Figure 5:**
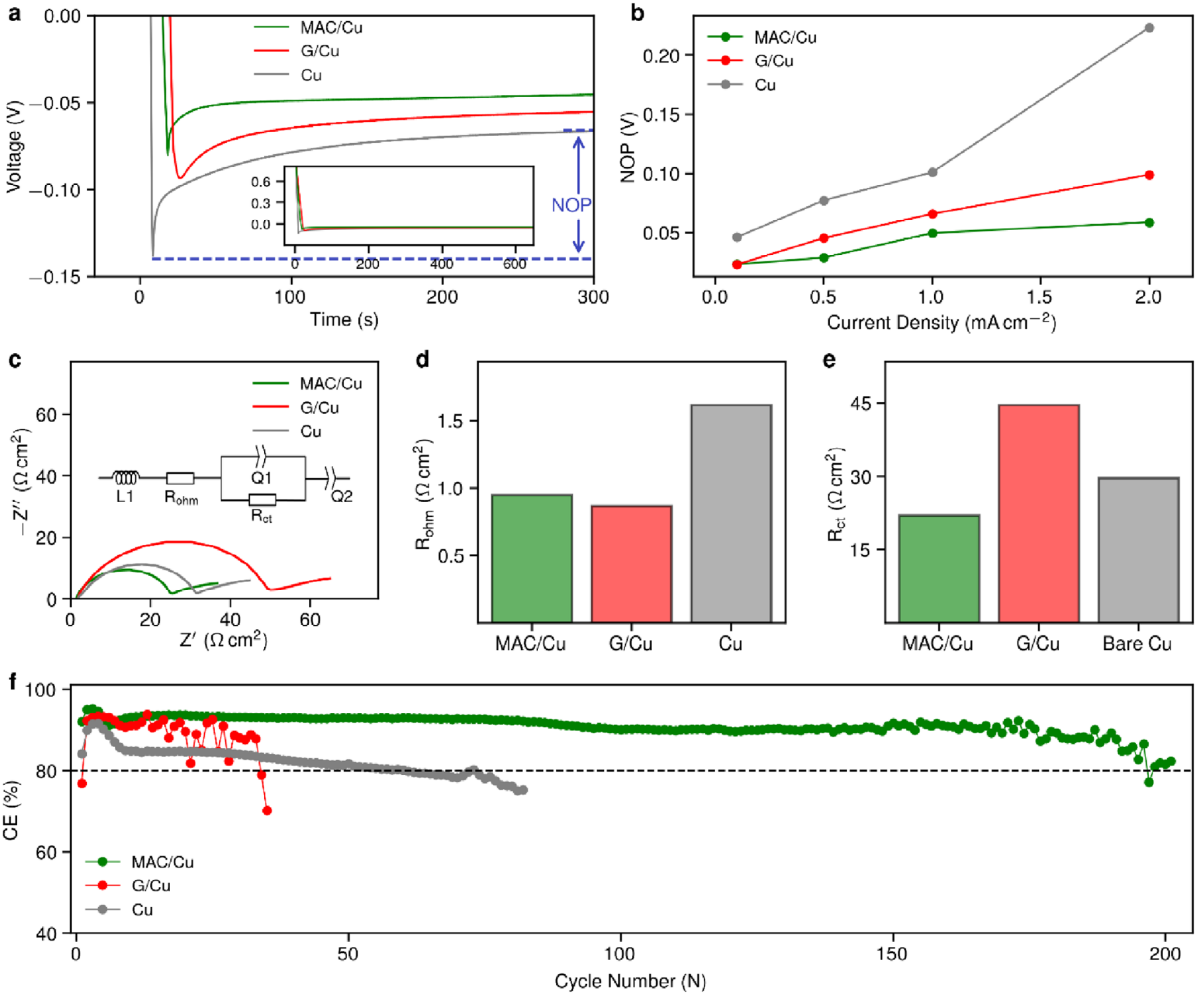
Electrochemical comparison of MAC/Cu, G/Cu, and Cu substrates in half‐cells vs metallic Li. a) Voltage‐time profiles recorded during Li nucleation at 0.5 mA cm^−2^ (inset: extended timescale). b) NOP vs current density. c) Nyquist plots after plating 0.5 mAh cm^−2^ at 0.5 mA cm^−2^; the inset shows the equivalent circuit model. d) Ohmic resistance (R_ohm_). e) Charge transfer resistance (R_ct_). f) CE over 200 plating/stripping cycles at 0.5 mA cm^−2^, 0.5 mAh cm^−2^.

To study Li nucleation dynamics, we conducted galvanostatic plating and electrochemical impedance spectroscopy (EIS) measurements in a coin cell battery configuration. The NOP is defined as the voltage difference between the lowest point reached during the onset of Li nucleation and the subsequent steady plating plateau in the voltage profile (indicated in blue in **Figure** **5**a). While this parameter is often attributed to the energy barrier for nucleus formation,^[^ ^66^, ^67^ ^]^ it should be recognized that the measured NOP reflects a convolution of multiple interfacial processes, including charge transfer across the electrode–electrolyte interface, ion transport in the electrolyte, and concurrent SEI formation during the early stages of plating.^[^ ^68^, ^69^ ^]^ A lower NOP, therefore, indicates that nucleation occurs more readily under the combined influence of these processes. The voltage‐time curves at the current density of 0.5 mA cm^−2^ for MAC on Cu foil (green curve), Cu (gray curve), and graphene (red curve) are shown in Figure 5a. At this current density, the NOPs were 28.9 mV for MAC/Cu, 45.5 mV for G/Cu, and 77.3 mV for Cu. As the current density increases (0.1–2 mA cm^−2^), NOP rises for all samples due to stronger kinetic limitations: interfacial charge transfer dominates at low current densities, whereas diffusion polarization and SEI dynamics become increasingly important at higher rates.^[^ ^70^ ^]^ But MAC/Cu consistently retained the lowest values (Figure 5b), supporting easier Li nucleation. The NOP of G/Cu was lower than that of bare Cu, in agreement with the report that graphene facilitates easier Li nucleation.^[^ ^29^, ^71^, ^72^, ^73^ ^]^ In contrast, the bare Cu surface possesses a different chemistry, potentially involving surface functionalization and higher activity toward parasitic electrolyte reactions (Figure 5d), which could lead to the higher NOP observed for Cu.

In addition to a low nucleation barrier, efficient electrochemical Li deposition also requires favorable interfacial charge transfer kinetics at the electrode–electrolyte interface. Although MAC is intrinsically in‐plane insulating, being only a monolayer thick, its out‐of‐plane electronic resistance at the MAC–Cu interface is sufficiently low to facilitate efficient charge transfer during Li ion reduction. To quantify MAC's effect on the total interfacial resistance, we conducted EIS measurements on asymmetric cells (MAC/Cu, G/Cu, and Cu vs Li metal) after an initial plating step (0.5 mA cm^−2^, 0.5 mAh cm^−2^). Representative Nyquist plots are shown in Figure 5c (Inset: the equivalent circuit model), with fitted values listed in Table S1 (Supporting Information). The extracted areal R_ohm_ were all close to 1 Ω·cm^2^ irrespective of the substrates (Figure 5d), confirming that the thin MAC coating does not significantly increase bulk cell resistance. Surprisingly, MAC/Cu exhibited markedly lower interfacialR_ct_ compared to G/Cu (Figure 5e), suggesting that MAC promotes the formation of an SEI with faster Li ion transport. The resulting rapid charge transfer further validates its exceptional electrochemical lithiophilicity and suitability for promoting uniform and efficient Li deposition. The uniform morphology, low NOP, and low R_ct_ observed on MAC/Cu are consistent with its enhanced cycling performance. We cycled at a 0.5 mA cm^−2^ current density and a 0.5 mAh cm^−2^ areal capacity; these conditions are widely used in mechanistic studies^[^ ^74^, ^75^, ^76^, ^77^, ^78^ ^]^ because they provide a regime where the interfacial properties of the electrode surface dominate the electrochemical response, allowing the influence of MAC's disorder‐induced lithiophilicity to be studied. While Cu and G/Cu cells faded below 80% CE after 61 and 34 cycles, respectively, MAC/Cu sustained 200 cycles, as shown in Figure 5f (for the striping capacity per cycle see Figure S14, Supporting Information). Cycling performance was verified using at least two cell replicates (Figure S15, Supporting Information). To indirectly assess the chemical stability of MAC after cycling, we performed a tape‐peeling experiment to gently remove the SEI from the surface, followed by Raman analysis of the exposed regions, which revealed an unchanged D/G intensity ratio (I_D_/I_G_ = 0.8) consistent with the as‐grown film (Figure S16, Supporting Information). While limited Li delamination was also observed in large‐area SEM of Li on MAC/Cu (Figure S12, Supporting Information), the effect is far less pronounced than on G/Cu, where extensive delamination of Li is evident (Figure S13, Supporting Information). The observed Li delamination likely originates from lateral pressure generated by adjacent Li domains during growth, which can mechanically disrupt adhesion and lift portions of the Li layer. It may also arise from incomplete MAC coverage or localized mechanical damage, which exposes small regions of Cu during cycling. To mitigate these effects, future optimization of MAC growth parameters should aim to achieve spatially uniform Li nucleation and homogeneous domain growth, thereby minimizing lateral stress accumulation between neighboring Li domains. Enhancing film continuity and interfacial bonding strength may further improve mechanical robustness. In parallel, exploring electrolytes or additives that reduce the SEI’s blocking effect on Li merging could help preserve interfacial integrity and extend cycle life.

## 3 Conclusion 

We have introduced disorder‐induced activation as a new and powerful design paradigm for functional 2D materials. This work demonstrates that intrinsic topological disorder can create a uniformly lithiophilic surface, providing a compelling alternative to conventional doping or alloying strategies. We show that a dopant‐free, single‐atom‐thick MAC film leverages its disordered sp^2^ network to generate a continuous landscape of strong Li binding sites (as low as −3.3 eV). This intrinsic affinity solves the fundamental scale‐mismatch problem of traditional coatings and translates directly to superior performance, including a low contact angle (31° for Li and <10° for LiTFSI), a reduced nucleation overpotential (29 mV at 0.5 mA cm^−2^), and a threefold improvement in cycling stability over bare copper. This study elucidates the fundamental mechanism by which atomic‐scale disorder activates metal affinity at carbon interfaces, laying the groundwork for future design of stable, lithiophilic coatings in next‐generation batteries. Beyond Li metal batteries, the principles of disorder‐engineered surface activation established here extend broadly to other electrochemical systems. The combination of atomic thinness and direct‐growth compatibility makes this strategy also promising for, e.g., stabilizing other alkali metal anodes, designing advanced electrocatalysts, and controlling thinfilm growth.

## 4 Experimental Section

### Preparation of Cu and G/Cu Substrates

Commercial Cu foils (35 µm thick) were cleaned by sequential sonication in acetone and isopropanol (10 min each), then annealed in a quartz tube furnace under 0.17 cm^3^ s^−1^ H_2_ flow at 0.17 mbar and 1030 °C for 2 h. Immediately after annealing, the Cu substrates were either characterized, used for MAC growth, or assembled into coin cells to minimize air exposure and prevent re‐oxidation. Single‐layer graphene on Cu (Graphene Institute Co., Ltd., Beijing) was used as received and characterized or cell‐assembled without further treatment.

### Synthesis of MAC

MAC was grown in a laser‐assisted chemical vapor deposition system equipped with an excimer laser (XeCl, λ = 308 nm, 50 Hz) and a remote inductively coupled plasma (ICP) source. A C_2_H_2_/Ar mixture (1:2 flow ratio) was introduced to keep the chamber pressure at 10^−3^ mbar, and the ICP power was set to the lowest value that sustained a stable glow discharge. The annealed Cu foil (2 × 6 cm), mounted on a resistively heated stage at 150 °C, wasirradiated at the center by the laser, which illuminated an area of ≈1 × 2 cm, with a fluence of ≈300 mJ cm^−2^. Film uniformity was verified by Raman line‐scan analysis (Figure S17, Supporting Information) and TEM sampling at multiple positions inside (Figure 1c) and outside (Figure S18, Supporting Information) the laser‐irradiated region, which showed indistinguishable amorphous structures. Growth times of 1, 2, and 4 min were investigated. All other parameters remained unchanged.

### Optimization of MAC

To evaluate the quality and uniformity of MAC coatings on Cu, corrosion tests were conducted on samples grown at a constant temperature but for 1, 2, and 4 min. The data (Figures S19 and S20, Supporting Information) indicate that a 4 min growth time produces the most continuous MAC film, as evidenced by the lowest corrosion density. Cycling data for 1, 2, and 4 min MAC samples (Figure S21, Supporting Information) further confirm that longer growth times improve stability, with enhanced film continuity mitigating localized failure and enabling better electrochemical reversibility. Based on these results, the 4 min MAC sample was selected for comparison with G/Cu and bare Cu in contact angle measurements and other electrochemical tests. Importantly, Raman spectra show that the three growth times yield the same I_D_/I_G_ ratio (Figure S22, Supporting Information), indicating that the degree of disorder remains constant. These results suggest that lithiophilicity is governed by intrinsic disorder rather than growth duration, while stability is determined by film continuity.

### Corrosion Test

Each substrate (Cu, G/Cu, and MAC/Cu) was immersed in a separate beaker of deionized (DI) water and maintained at 60 °C for 4 h. Following this thermal exposure, samples were removed, dried under a gentle N_2_ stream, and examined by SEM (FEI Verios 460). for signs of surface corrosion.

### Sample Preparation for Raman Spectroscopy

For both films, the carbon layer on the backside of the Cu foil was first removed by 50 W Ar plasma for 5 min. MAC: A polymer‐free wet transfer was performed. The Cu was floated on 1 wt.% ammonium persulfate (APS) until fully etched; the free‐floating MAC was scooped onto Si/SiO_2_ wafers, rinsed twice in DI water (2 h each time), air‐dried, and baked at 120 °C for 30 min. Graphene: a PMMA‐assisted transfer was used. A 4% PMMA A4 solution (in anisole) was spin‐coated at 4000 rpm for 60 s and baked at 180 °C for 2 min. After etching the Cu in 1 wt.% APS, the PMMA/graphene stack was rinsed twice in DI water (2 h each time) and scooped onto Si/SiO_2_ wafers, dried, baked at 120 °C for 30 min, and the PMMA was removed in acetone (2 h) followed by an isopropanol rinse.

### Raman Spectroscopy

Raman spectra were collected at room temperature using a WITec Alpha 300R microscope with a 532 nm excitation laser. Measurements were carried out using a laser power of 0.2 mW and a total accumulation time of 30 s. An open‐source Rampy package was used to remove the background in the spectra collected from the MAC on Cu foil. Particularly, the asymmetric least squares correction method was used to calculate the background curve.

### Sample Preparation for TEM

For both films, the carbon layer on the back side of the Cu foil was first removed by 50 W Ar plasma for 5 min. MAC: the polymer‐free transfer was repeated, but the floating film was scooped directly onto TEM grids, rinsed, air‐dried, and baked at 120 °C for 30 min. Graphene: a PMMA‐free scoop‐up method was used. TEM grids were placed face down on G/Cu, and the assembly was floated on 1 wt.% APS, and after Cu etching, the grid/graphene stack was rinsed twice in DI water (2 h each time), air‐dried, and baked at 120 °C for 30 min.

### TEM

TEM imaging was performed on a noncommercialized JEOL ARM60 microscope equipped with a Schottky field emission gun, a JEOL double Wien filter monochromator, double delta correctors, and a Gatan OneView camera. A slit of 2.8 µm was used for energy filtering, enabling a spatial resolution of 1.1 Å with a beam current of ≈20 pA. The microscope operated at 60 kV. STEM imaging was carried out in a JEOL 2100F microscope equipped with a Delta probe corrector, which corrects the aberration up to fifth order, resulting in a probe size of 1.0 Å. The convergent angle for illumination was ≈35 mrad, with a collection detector angle ranging from 45 mrad to 200 mrad. A JEOL heating holder was used in all experiments to heat the sample up to 700 °C during imaging.

### Cross‐Sectional TEM

For the preparation of cross‐sectional TEM samples Helios 450S Focused Ion Beam (FIB) system was used. MAC samples grown on Cu were capped with a PtPd protection layer, and further, a thin layer of Pt was deposited in situ within the FIB. A site‐specific lamella was then extracted using a standard lift‐out procedure with Gallium (Ga^+^) ions at 30 kV. The lamella was thinned to electron transparency using progressively lower ion currents, followed by a final low‐energy cleaning step at 2 kV to remove surface amorphization and Ga^+^ implantation. High‐resolution cross‐sectional imaging was performed on a ThermoFisher Titan TEM/STEM operated at 300 kV and equipped with a Gatan OneView camera.

### STM and STS

Low‐temperature STM/STS measurements were performed in an Omicron low‐temperature STM (≃4.2 K) under ultra high vacuum conditions (≃5 × 10^−11^ mbar). The STM topography was measured in constant current mode using a chemically etched tungsten (W) tip. Prior to the actual measurements, the W tip was calibrated on Au (111) by voltage pulses or controlled indentations into the surface until the herringbone without artifacts and atomic resolution was achieved followed by measuring the Shockley surface states of Au (111).

### Contact Angle Measurements (Li & LiTFSI)

All measurements were carried out in an argon‐filled glovebox to avoid surface contamination. For molten Li, a fresh Li droplet was prepared by gently scraping away its native oxide/carbonate layer until a metallic luster appeared. The foil was then folded and lightly compressed into a roughly spherical pellet, which was placed on a substrate heated to 250 °C using a temperature‐controlled hot plate. Prior to use, the Cu, G/Cu, and MAC/Cu substrates were pre‐annealed at 250 °C for 10 min to eliminate residual surface moisture. Still images of the molten droplet were captured from a side view for analysis. For melted LiTFSI, the powder was directly deposited onto the preheated substrate and melted at 250 °C. Still images were captured at ≈30° above the horizontal (relative to the substrate plane) to enhance visibility of the contact angle. The intrinsic chemical stability of graphene and MAC further reduces the likelihood of surface oxidation under inert conditions, ensuring reliable wetting measurements.^[^ ^49^ ^]^

### Coin Cell Assembly and Measurements

Substrates were cut to 14 mm in diameter and assembled into CR2032 cells using the test substrate as the working electrode, Li metal as both counter and reference, two stainless‐steel spacers, and one stainless‐steel spring. Each cell was filled with 70 µL of electrolyte (1 m LiTFSI in a 1:1 (v/v) mixture of 1,3‐dioxolane and dimethoxyethane containing 1 wt.% LiNO_3_). This corresponded to an electrolyte‐to‐capacity ratio of ≈90 mL Ah^−1^ for the tested areal capacity (0.5 mAh cm^−2^). This ratio was kept consistent across all samples; minor variation (≤10%) may appear due to slight electrolyte loss during the crimping process or small dispensing inaccuracy, which is typical for coin cell assembly but does not affect cycling stability under this excess‐electrolyte condition. After assembly, cells were rested for 10 h before testing to ensure complete wetting. All electrochemical tests are done at room temperature. After Li deposition, cells were disassembled in the glovebox; the foils were rinsed with dimethoxyethane, dried on a hot plate at 70 °C for 10 h, and then the Li morphology was examined by SEM.

### NOP Analysis

NOP was determined from the voltage–time curves recorded during galvanostatic Li plating. The minimum potential at the onset of nucleation (E_nuc_) marks the formation of critical Li clusters. As plating continues, the voltage rises to a quasi‐steady growth plateau (E_growth_) where the nuclei expand. The difference ΔE = E_growth_– E_nuc_ defines the NOP, isolating the intrinsic energy barrier for nucleus formation from additional polarization or resistive contributions during deposition.

## 5 Computational Section

### MAC Structure Generation

The MAC used in the single Li atom adhesion calculation was generated through high‐temperature molecular dynamics (MD) annealing of a polycrystalline initial configuration. The initial polycrystal structure containing 458 C atoms was defined in a square supercell of dimensions *L_x_* = 35 Å, *L_y_* = 35 Å with individual crystalline regions randomly oriented and bounded by the edges of a Voronoi diagram. The amorphicity of MAC is effectively controlled by the density of the randomly distributed Voronoi centers, where a density of 0.012 centers per Å^2^ yielded ring distributions comparable to the experiment (mismatch < 6% for 5‐, 6‐, 7‐ and 8‐membered rings). The subsequent annealing was first confined to the 2D plane and was performed within the canonical (NVT) ensemble (friction coefficient τ = 1 ps).^[^ ^79^, ^80^ ^]^ Temperatures were applied up to 8000 K followed by a cooling down of the sample to below 300 K with a cooling rate of ≈4 fs K^−1^. A second annealing step allowed for out‐of‐plane relaxation, albeit at a lower maximal temperature of 2000 K, and was followed by structural minimization. The simulations were performed using the classical molecular dynamics code LAMMPS^[^ ^81^ ^]^ with CH.airebo^[^ ^82^ ^]^ force field coefficients. A smaller amorphous carbon monolayer containing 128 C atoms was generated for the interfacial adhesion calculation following the quench from the melt method described in the earlier publication.^[^ ^49^ ^]^

### DFT Calculations

The calculations of adhesion energies of single Li atoms were performed with the first principles code SIESTA (v4.1.5).^[^ ^83^, ^84^ ^]^ The localized spin‐polarized DZP basis set was used with the mesh cutoff set to 250 Ry. Furthermore, the DRSLL van der Waals density functional,^[^ ^85^, ^86^ ^]^ together with Troullier‐Martins GGA pseudopotentials^[^ ^87^ ^]^ for Li and carbon, was used. DFT calculations of interfacial adhesion energies were performed using a planewave basis set and the Projector Augmented Wave formalism^[^ ^88^, ^89^ ^]^ as implemented in the Vienna Ab Initio Simulation Package.^[^ ^90^, ^91^, ^92^, ^93^ ^]^ The Perdew–Burke–Ernzerhof^[^ ^94^ ^]^ exchange‐correlation functional was used in conjunction with the Grimme D3 van der Waals correction,^[^ ^95^ ^]^ and the kinetic energy cutoff was set to 520 eV. In both calculations, a vacuum layer of > 20 Å was introduced in the out‐of‐plane direction for all slab calculations to prevent spurious interactions, and all structures were relaxed with a maximum force tolerance of 0.05 eV Å^−1^.

### Li‐Atom Adsorption Calculations

The adhesion energy of a single Li atom was calculated for the MAC model, graphene, and the Li(111) surface. In the case of MAC, Li binding sites were proposed to be at a ≈2Å offset along the normal axis from each of the 228 distinct ring centers of the sample. The site‐dependent adhesion energies were calculated as

(3) $$E_{a}=\;E_{Li@MAC}-E_{MAC}-E_{Li}$$

where *E*_*Li*@*MAC*_ is the energy of the relaxed Li@MAC structure, *E_MAC_* is the energy of the relaxed MAC structure without Li, and *E_Li_* is the energy of a single Li‐atom in vacuum. The case of graphene is analogous, and used a *L_x_* = 17.04 Å, *L_y_* = 12.30 Å supercell with 80 carbon atoms to prevent Li–Li interactions resulting in a single characteristic adsorption energy (for the equivalent 6‐membered rings). Finally, Li‐adsorption on the Li(111) surface was modeled using a 6‐layer Li slab with a hexagonal 3 × 3 supercell (a = $3\times \sqrt{2}\times 3.65$ Å, c = 40 Å). A 1 × 1 × 1, 9 × 9 × 1 and 10 × 10 × 1 Monkhorst‐Pack *k*‐grid was used for the MAC, graphene, and Li slab calculations, respectively.

### Contact Angle Calculations

The contact angle is determined using Young's equation,^[^ ^96^ ^]^

(4) $$\gamma_{S,V}-\gamma_{S,Li}=\gamma_{Li,V}\cos\left(\theta \right)$$

where γ_*S*,*V*_, γ_*S*,*Li*_ and γ_*Li*,*V*_ are the surface tensions at the substrate‐vacuum, substrate‐Li and Li‐vacuum interfaces, respectively. It is calculated as

(5) $$\gamma_{Li,V}=\;\frac{1}{2A}\left(E_{Li,slab}-E_{Li,bulk}\right)$$

(6) $$\begin{array}{ccc} \gamma_{S,V}-\gamma_{S,Li} & = & -\frac{1}{A}\left(E_{SLi,slab}-E_{S,slab}-E_{Li,slab}\right)-\gamma_{Li,V}\;\\ & = & -E_{SLi,adh}-\gamma_{Li,V} \end{array}$$

Here, *A*  is the contact area for a single interface, $E_{Li,bulk}$ is the energy of Li atoms in the bulk, while *E*_*Li*,*slab*_, $E_{S,slab}$ and *E*_*SLi*,*slab*_ are the energies of a slab of Li, the bare substrate, and the combined substrate‐Li slab heterostructure, obtained from DFT relaxed geometries. The MAC/Li heterostructure was constructed by placing the MAC model (strain < 2.5%) atop a 4 × 4 supercell of a 6‐layer Li(111) slab. Similarly, the graphene/Li heterostructure was generated by placing (strain < 2.5%) a 2 × 2 graphene supercell with 8 C atoms on a 1 × 1 Li(111) slab. For all simulations, lattice constants were fixed to the bulk Li value of a=3.40Å. A 1×1×1, 40×40×1, and 40×40×40 Monkhorst‐Pack k‐grid were used for the MAC, graphene, and Li bulk calculations, respectively.

## Conflict of Interest

The authors declare no conflict of interest.

## Author Contributions

L.S., H.Z., A.K.G. and R.Y. contributed equally to this work. B.Ö. initiated and supervised the project. L.S. performed contact angle and coin cell measurements. H.Z. performed DFT calculations. A.K.G. coordinated the experimental workflow and contributed to data analysis. R.Y. coordinated DFT calculations and contributed to data analysis. O.V.Y. supervised the DFT calculations. R.SK, R.S., Z.J.T. and B.W. conducted STM imaging. H.Z., K.V.I., A.A.A., A.S., C.M.O. synthesized the materials and performed sample characterizations. R.M., T.M.T. and N.Q. supported materials synthesis and contributed to conceptual development of the work. J.L. and K.S. performed HRTEM imaging. L.S., H.Z., A.K.G., R.Y., S.L., S.A., C.‐T.T. and B.Ö. wrote the manuscript. All authors discussed the results and contributed to the manuscript.

## Supporting information



Supporting Information

## Data Availability

The data that support the findings of this study are available from the corresponding author upon reasonable request.
