# Supplementary material for: Monolayer Amorphous Carbon: Unlocking Disorder‐Induced Lithiophilicity
Source: Adv Sci (Weinh). 2025 Nov 25;13(3):e16490. doi: 10.1002/advs.202516490 (PMC12806237; doi:10.1002/advs.202516490)
Supplement: Supplementary file 1 — Supporting Information [file ADVS-13-e16490-s001.docx]

Supporting Information

**Monolayer Amorphous Carbon: Unlocking Disorder-Induced Lithiophilicity**

*Lu Shi, Hanning Zhang, Artem K. Grebenko, Ruslan Yamaletdinov, Rejaul SK, Ranjith Shivajirao, Zheng Jue Tong, Sergey Luchkin, Hongji Zhang, Konstantin V. Iakoubovskii, Alena A. Alekseeva, Andrei Starkov, Carlo M. Orofeo, Junhao Lin, Kazutomo Suenaga, Chee-Tat Toh, Remi Mahfouz, Talah M. Tayeb, Nada Qari, Stefan Adams, Bent Weber, Oleg V. Yazyev, Barbaros Ӧzyilmaz**


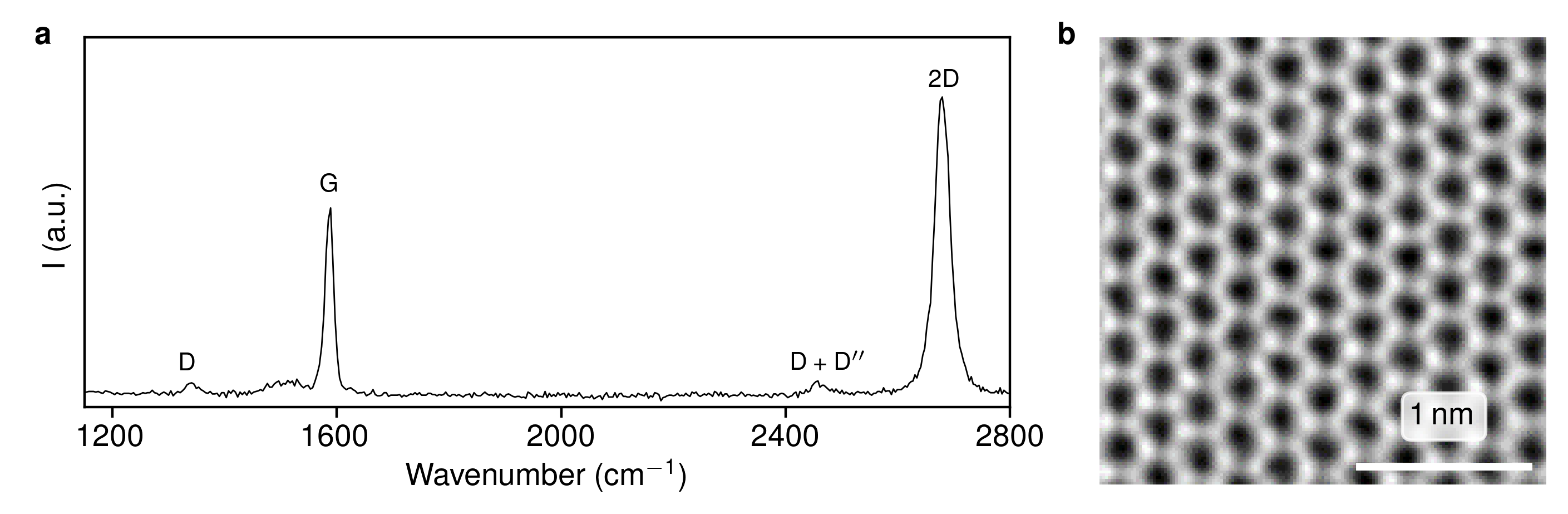


**Figure S1.** (a) Raman spectrum of transferred graphene, showing strong G and 2D peaks at 1580 and 2680 cm⁻¹, respectively, and a weak D band. (b) TEM image of freestanding graphene with perfect hexagonal lattice.


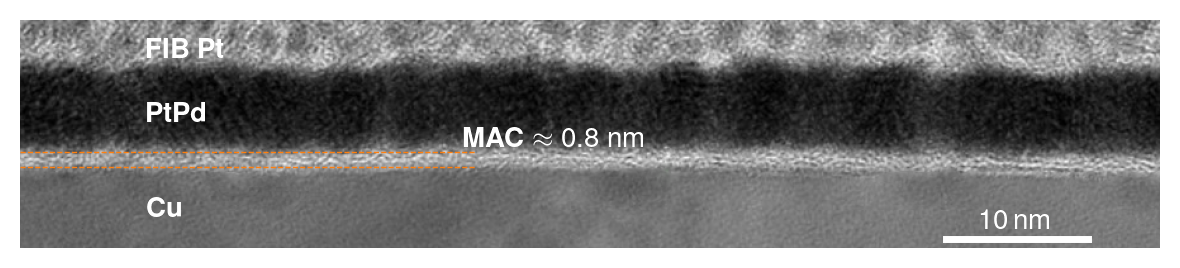


**Figure S2.** Cross-sectional TEM image at the PtPd-MAC-Cu interface with a uniform MAC thickness of 0.8 nm.


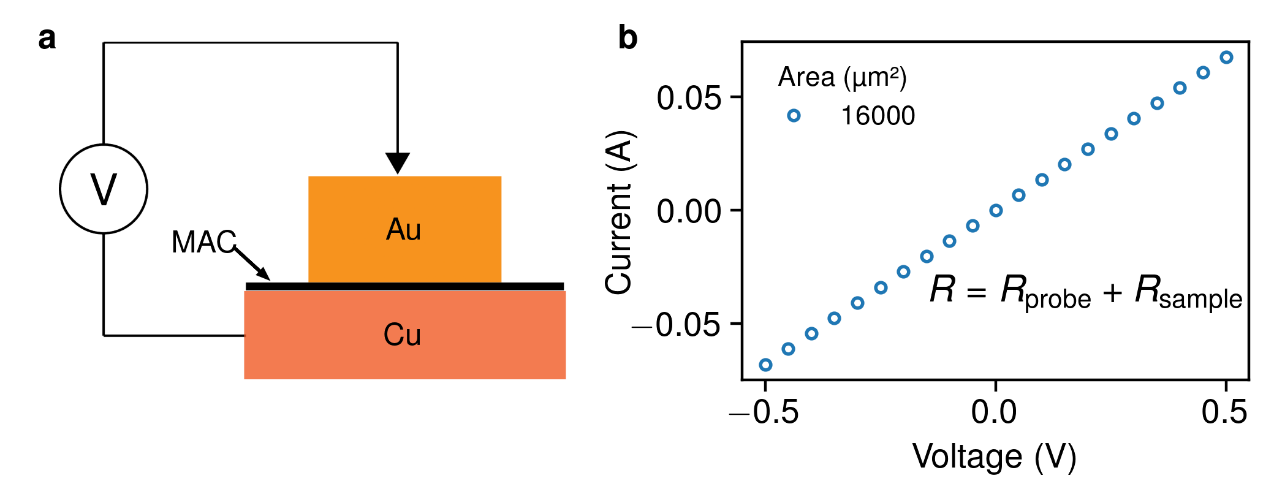


**Figure S3.** Out-of-plane conductivity measurement of MAC. (a) Schematic of the two-probe configuration used to measure the I–V characteristics, with Au serving as the top contact and Cu as the bottom electrode. (b) I–V curve showing a linear response, indicating ohmic behavior across the Au/MAC/Cu stack.

To evaluate the out-of-plane resistance of the MAC film, Au/MAC/Cu junctions were fabricated by thermally evaporating 60 nm Au pads of various areas onto as-grown MAC/Cu substrates. The measurement configuration (Figure S3a) employed a two-probe geometry, where a voltage was applied between the Au pad and Cu substrate using a probe station, and the resulting current was recorded. This setup measures the total resistance $R=R_{\text{probe}}+R_{\text{sample}}$, combining the probe contact resistance and the intrinsic out-of-plane resistance of the MAC layer.

An example I–V curve for a contact area of 1.6 × 10⁴ µm² (Figure S3b) exhibits linear (ohmic) behavior, confirming good electrical coupling across the Au/MAC/Cu stack. The total resistance from the linear fit is $R=7.38\text{ }\Omega$. After subtracting the probe contact resistance ($R_{\text{probe}}=7.01\text{ }\Omega$, determined from area-dependent fitting in Figure S4), the specific out-of-plane resistance of the MAC layer was extracted to be 6.53×10^-5^ Ω·cm^2^.


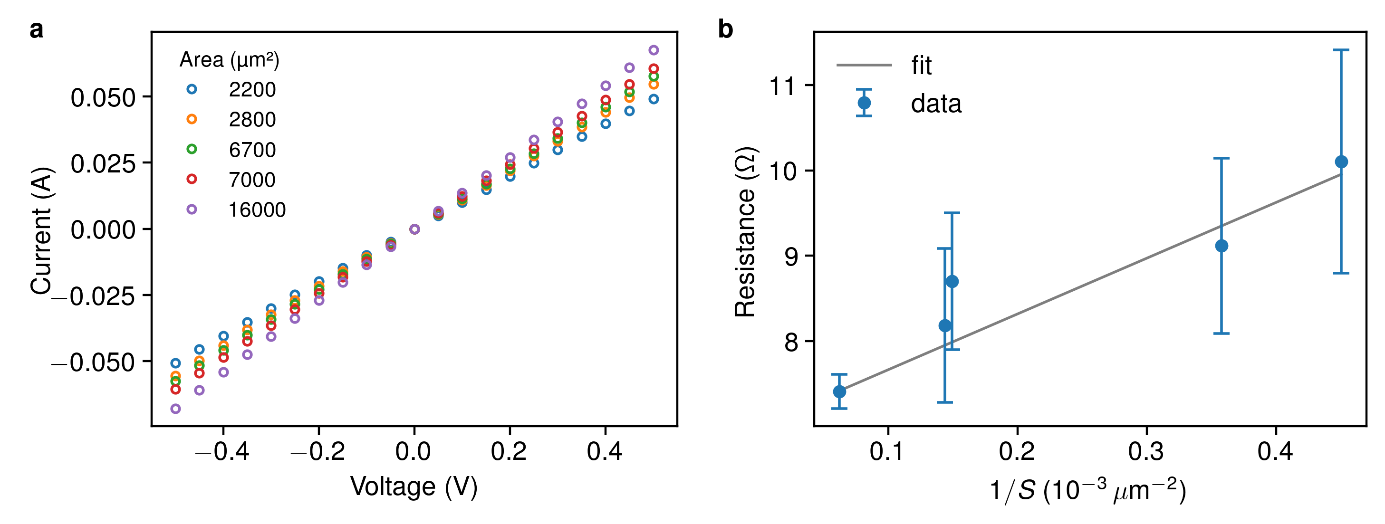


**Figure S4.** Determination of probe contact resistance during the out-of-plane conductivity measurement of MAC. (a) Current–voltage (I–V) characteristics of Au/MAC/Cu junctions measured for Au pads of different contact areas, showing linear (ohmic) behavior. (b) Extracted total resistance as a function of Au contact area. The data were fitted using $R=R_{c}+\rho d/S$, where $\rho$ is the out-of-plane resistivity of MAC, $d$ is the thickness, and $S$ is the contact area. The intercept of the fit yields $R_{c}$, representing the probe contact resistance, while the slope corresponds to the contribution from the MAC film.

To accurately quantify the out-of-plane resistance of MAC, we first determined the probe contact resistance ($R_{c}$). Au pads of varying contact areas (2,200–16,000 µm²) were thermally deposited onto the as-grown MAC/Cu substrate to form Au/MAC/Cu stacks. I–V characteristics were measured using a probe station under ambient conditions. The extracted total resistance ($R_{\text{tot}}$) decreased with increasing contact area, consistent with the relation $R_{\text{tot}}=R_{c}+\rho d/S$, where $\rho$is the out-of-plane resistivity of MAC, $d$its thickness, and $S$the contact area. Fitting the experimental data yielded a probe contact resistance of $R_{c}=7.01\text{ }\Omega$.


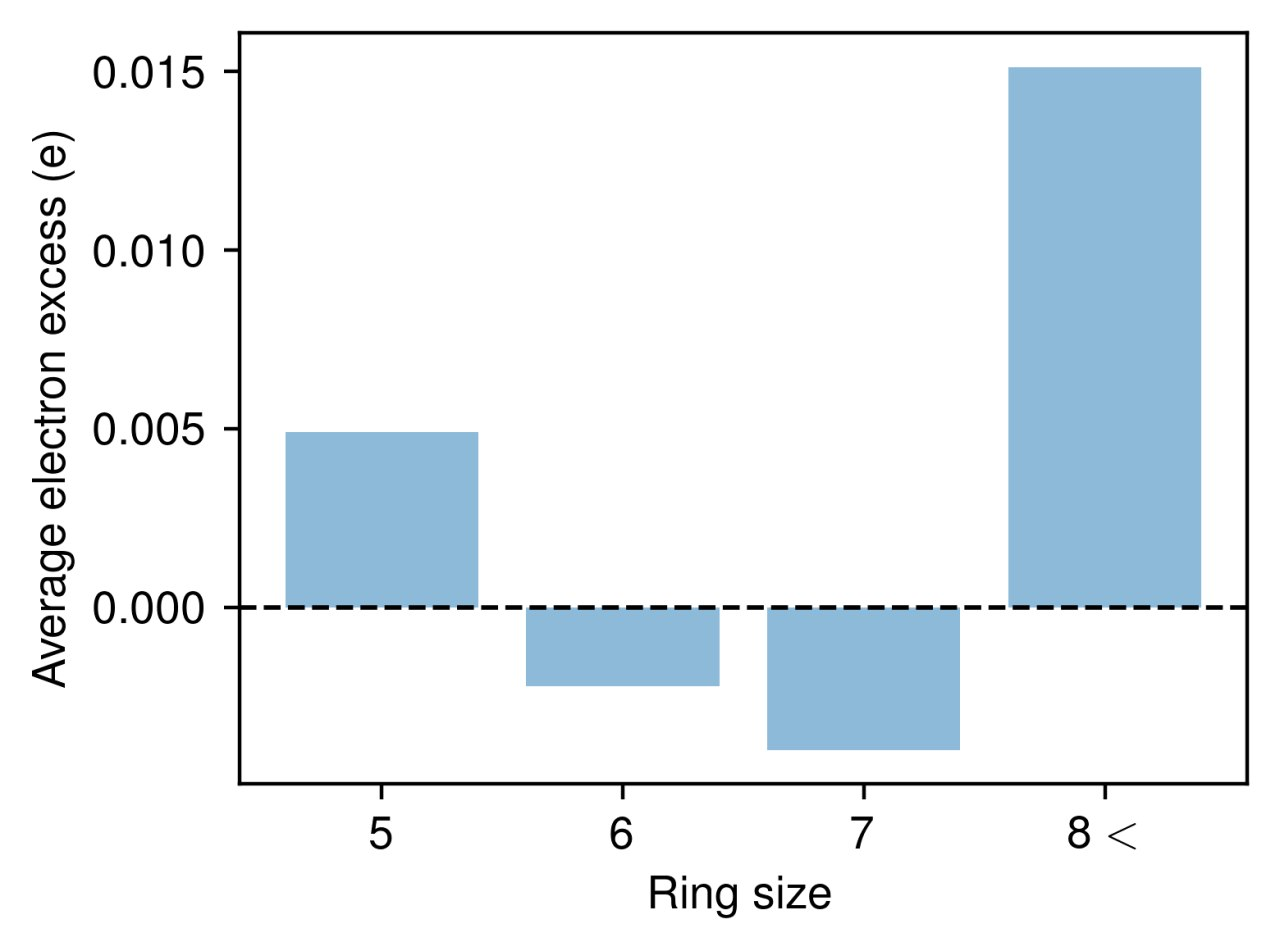


**Figure S5.** Average electron excess for different ring sizes. Hexagons and heptagons exhibit an electron deficit, while pentagons and octagons show an electron excess. The electron deficit for hexagons in particular shows that the introduction of amorphous regions in MAC is a viable strategy of reducing electronegativity and increasing Li adhesion.


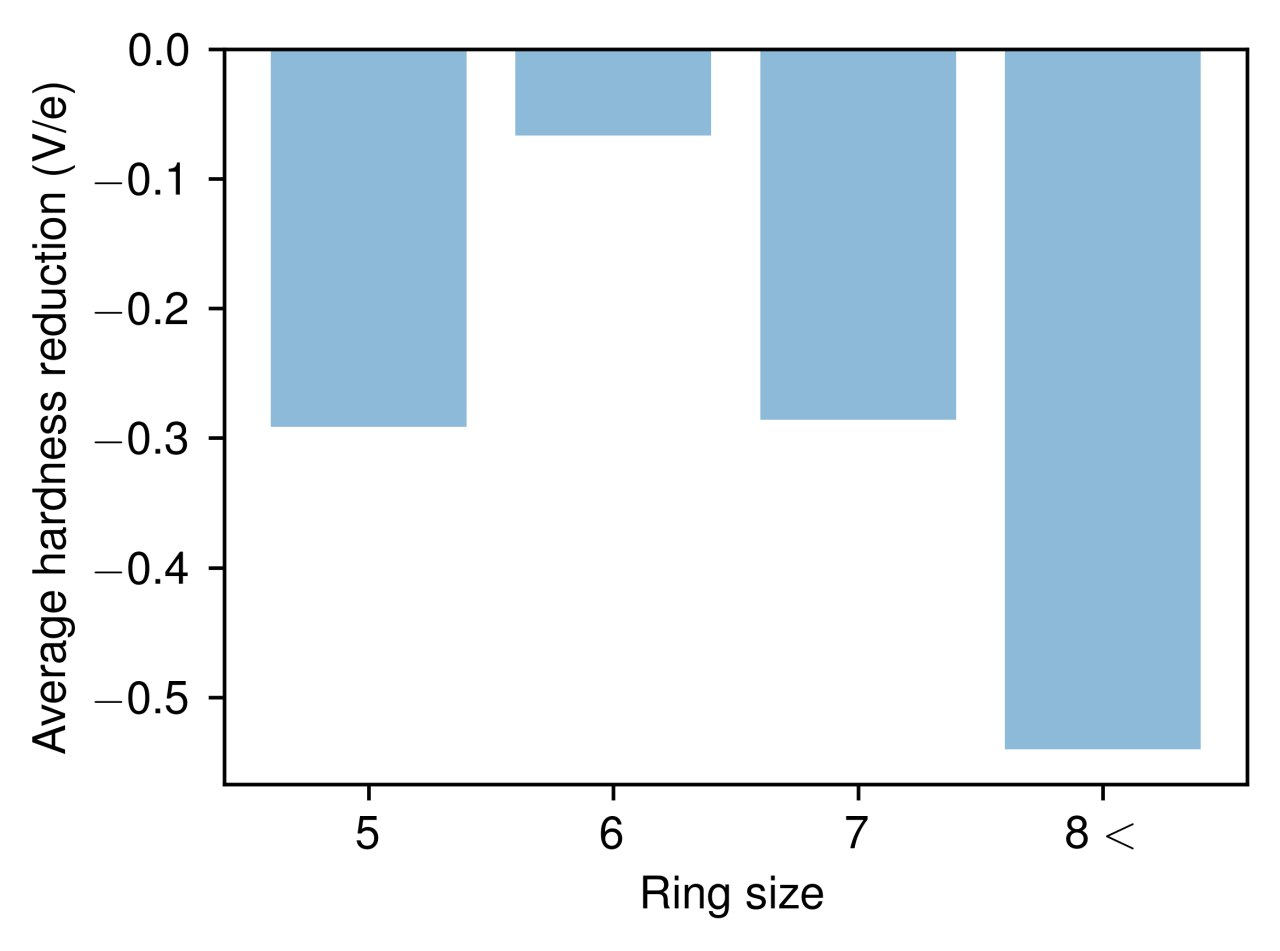


**Figure S6.** Average hardness correction due to the screening effect across different ring sizes.


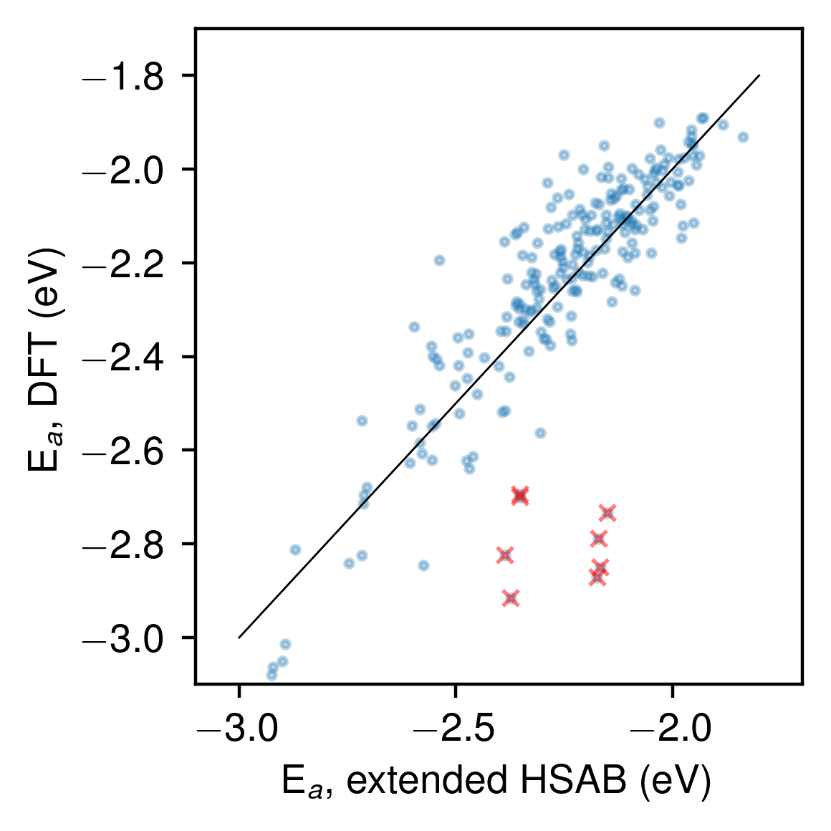


**Figure S7.** Error analysis shows only 9 cases for which the HSAB estimated adhesion energy strongly (>0.3 eV) deviates from the DFT-calculated one, and all but one case is due to an underestimation of the binding energy (marked as red crosses). We argue that this underestimation is due to rare covalent bonding (Figure S8) with carbon atoms. Interestingly, only 3 out of 458 carbon atoms show sufficiently small Li-C bond length and underestimation of the HSAB energy indicative of covalent bonding.


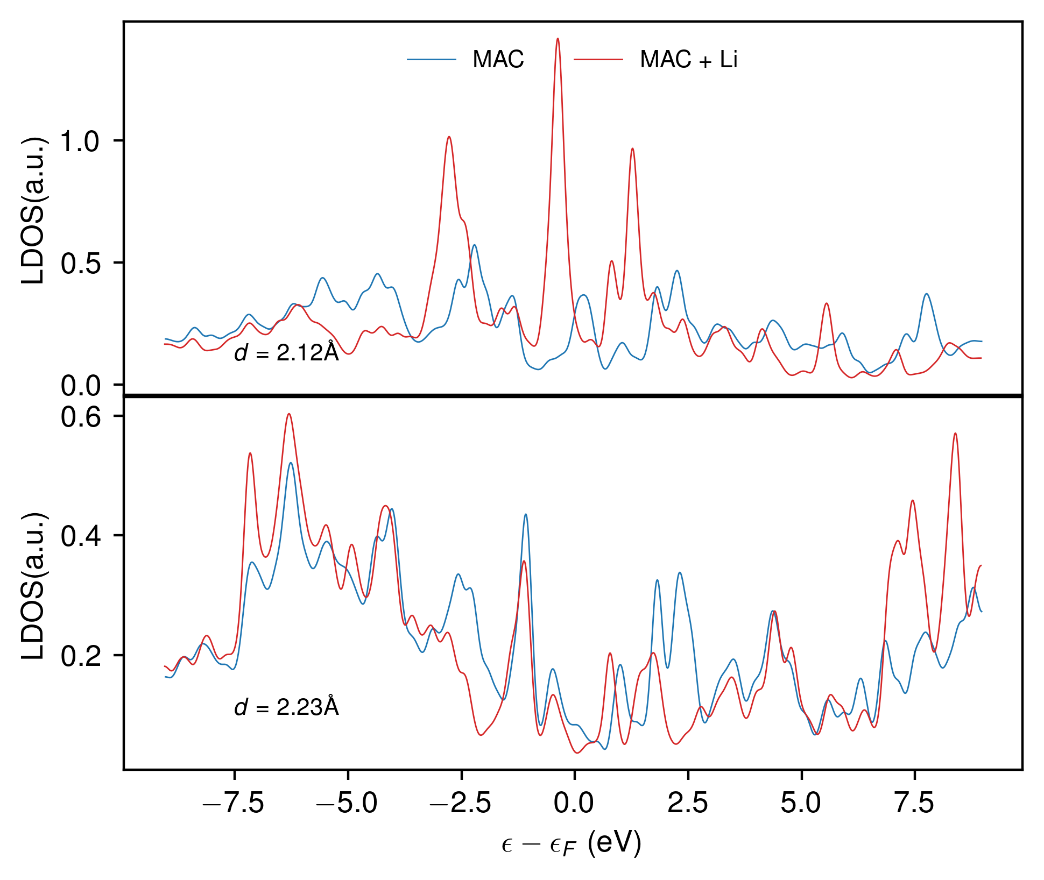


**Figure S8.** LDOS for one of the outlier Li sites (Figure S7), with significant HSAB underestimation of the adhesion energy. The LDOS of the closest carbon site is notably perturbed by the Li atom with new states emerging at the Fermi level-consistent with covalent bond formation.


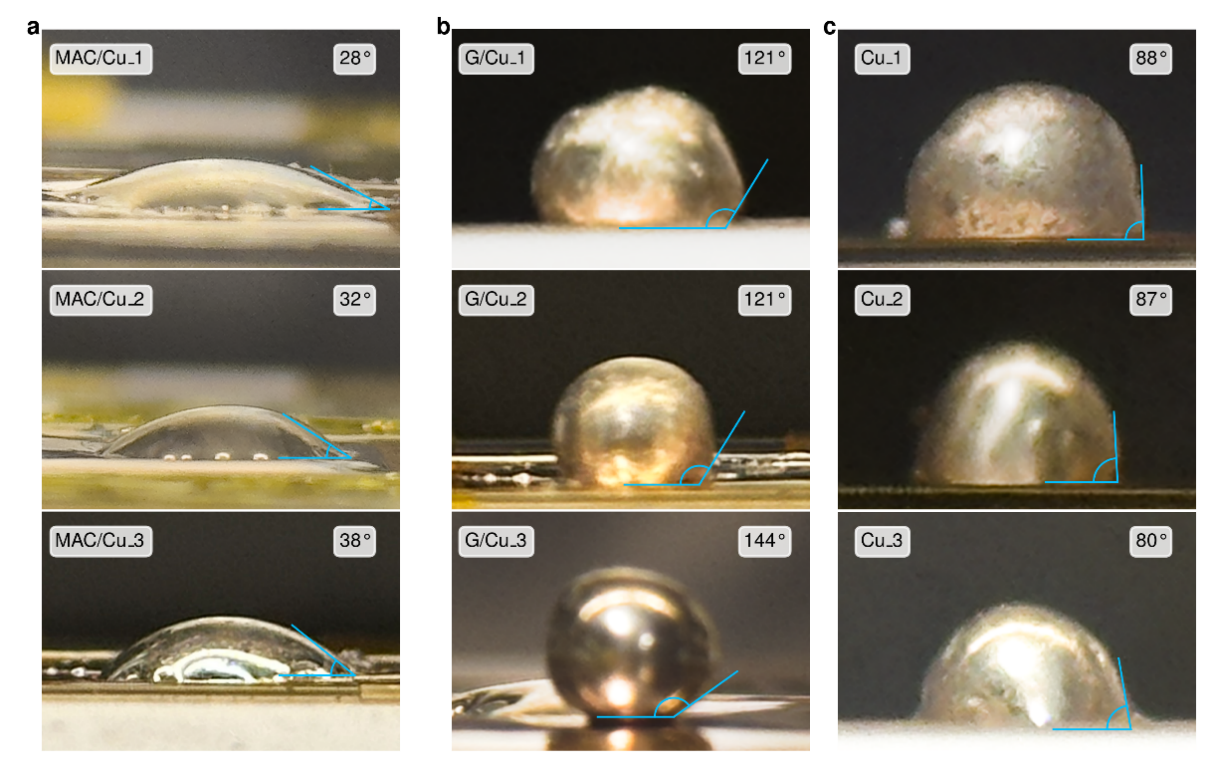


**Figure S9.** Additional optical image data sets of the contact angle measurements on different substrates: (a) Li on MAC/Cu, (b) Li on G/Cu and (c) Li on Cu.


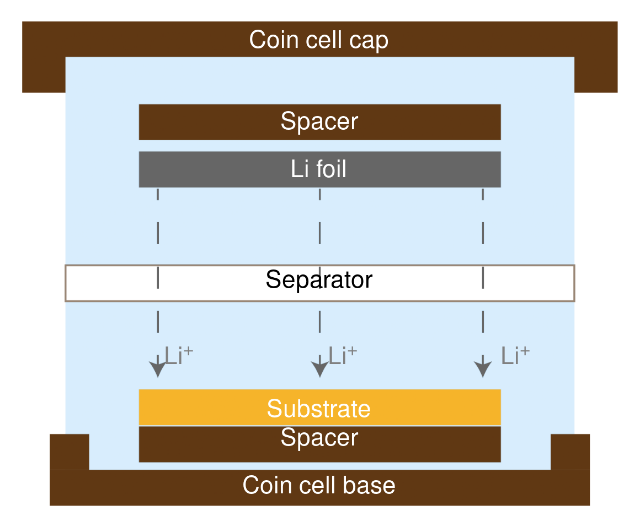


**Figure S10.** Schematic of the coin cell structure.


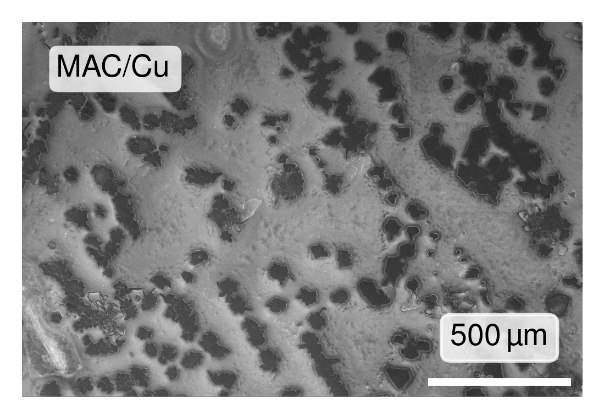


**Figure S11.** SEM image of the Li morphology on MAC/Cu on a large scale.

In addition to the localized-view SEMs in the main text, large-area SEM imaging of MAC/Cu further confirms the uniformity of Li nucleation and growth across millimeter‐scale regions. Even at low magnification, individual Li islands retain a remarkably consistent and flattened morphology.


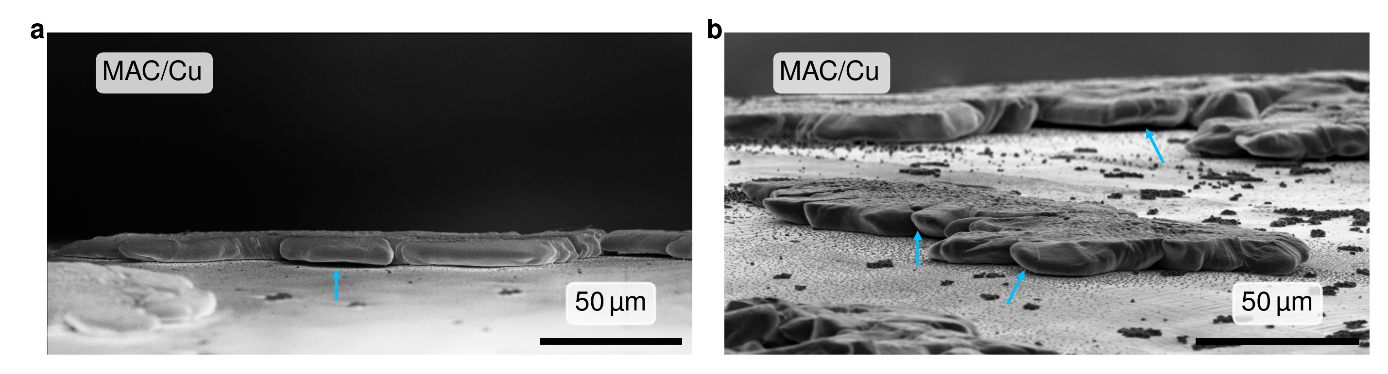


**Figure S12.** Cross-sectional SEM images of Li deposited on MAC/Cu. Blue arrows indicate regions where the plated Li does not adhere well to the surface.


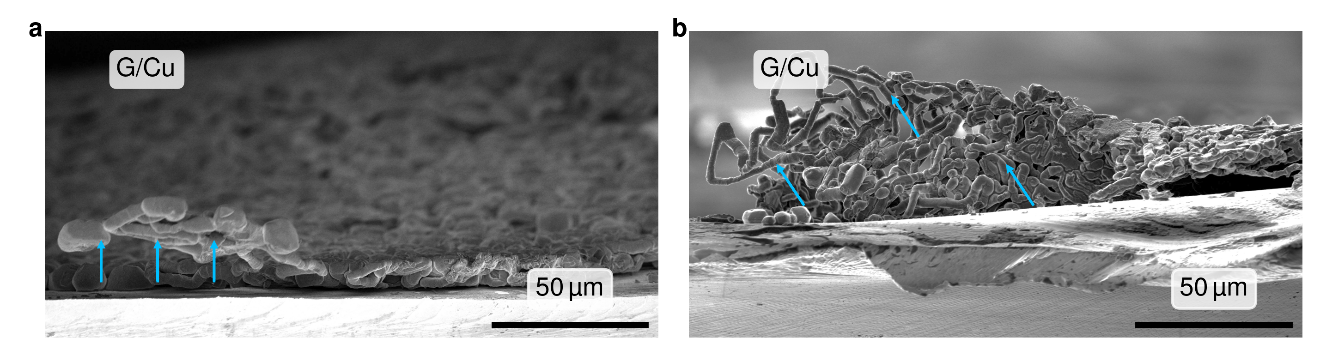


**Figure S13.** Cross-sectional SEM images of Li deposited on G/Cu. Blue arrows indicate regions where the plated Li has started to lift off from the surface.


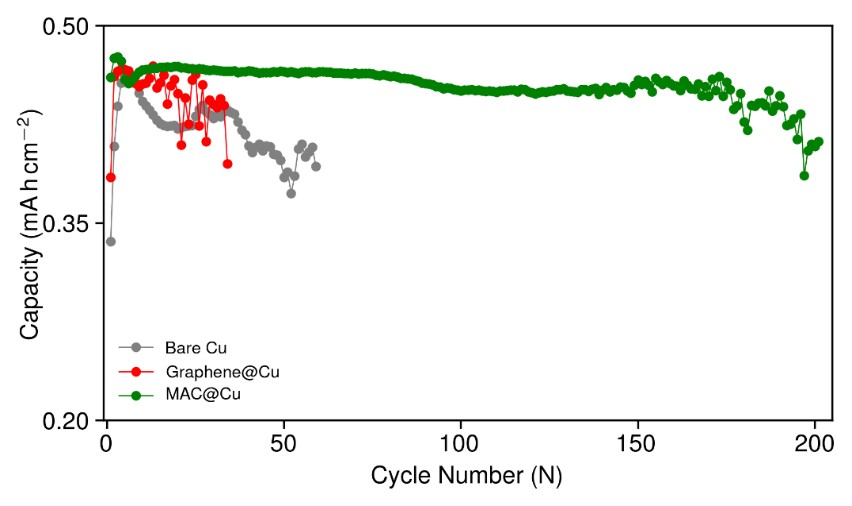


**Figure S14****.** Capacity density versus cycle number for bare Cu (grey), G/Cu (red) and MAC/Cu (green)..


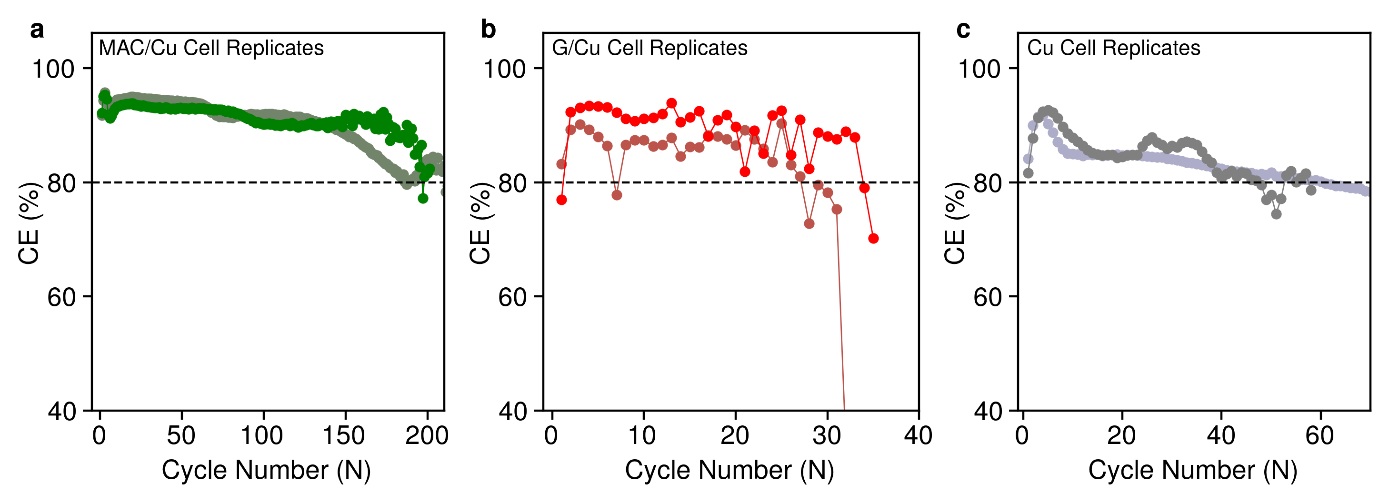


**Figure S15.** CE of replicate half-cells plated/stripped at 0.5 mA cm⁻²: (a) MAC/Cu, (b) G/Cu, (c) Cu.


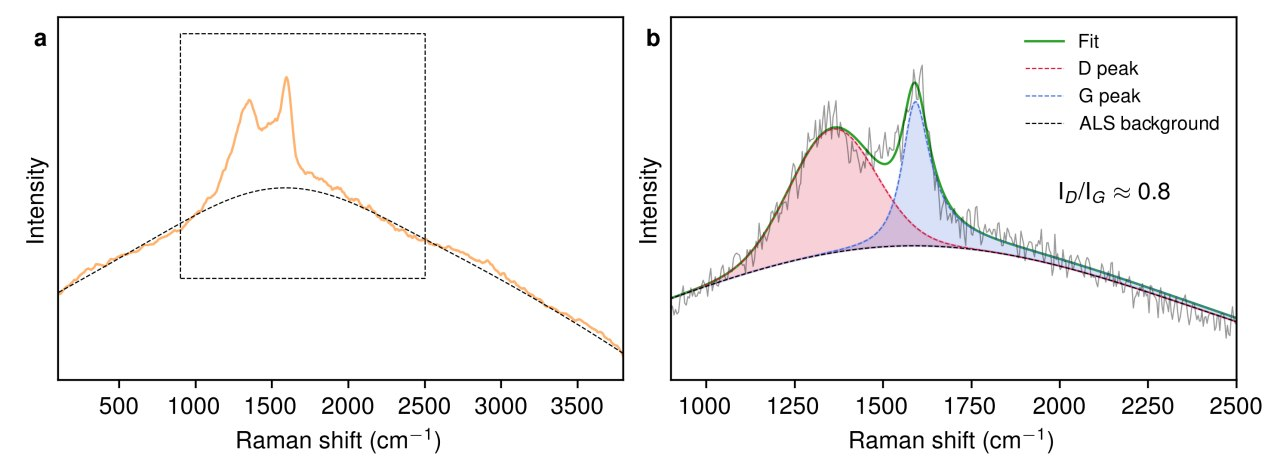


**Figure S16.** Post-cycling Raman analysis of the MAC film after SEI removal. (a) Raman spectrum of the cycled MAC/Cu electrode after peeling off the SEI using adhesive tape. (b) Fitted Raman spectrum of the same region showing the D and G bands with an intensity ratio (I_D_/I_G_ =0.8), same to the as-grown MAC.

To assess the chemical stability of MAC after electrochemical cycling, we performed a tape-peeling experiment to gently remove the SEI from the surface, followed by Raman analysis of the exposed regions. As shown in Figure S16, clear MAC spectroscopic features were detected in some areas with an unchanged D/G intensity ratio (I_D_/I_G_=0.8) relative to the as-grown film, indicating that the disordered sp² framework remained intact after cycling. In other regions, the MAC signal was absent, likely because the peeling process partially detached the ultrathin MAC layer itself. These results suggest that the underlying MAC film largely preserves its structural integrity during cycling. However, given the inherently invasive nature of the peeling process, it is possible that the procedure locally modified or disrupted the MAC film, influencing the detected signal.


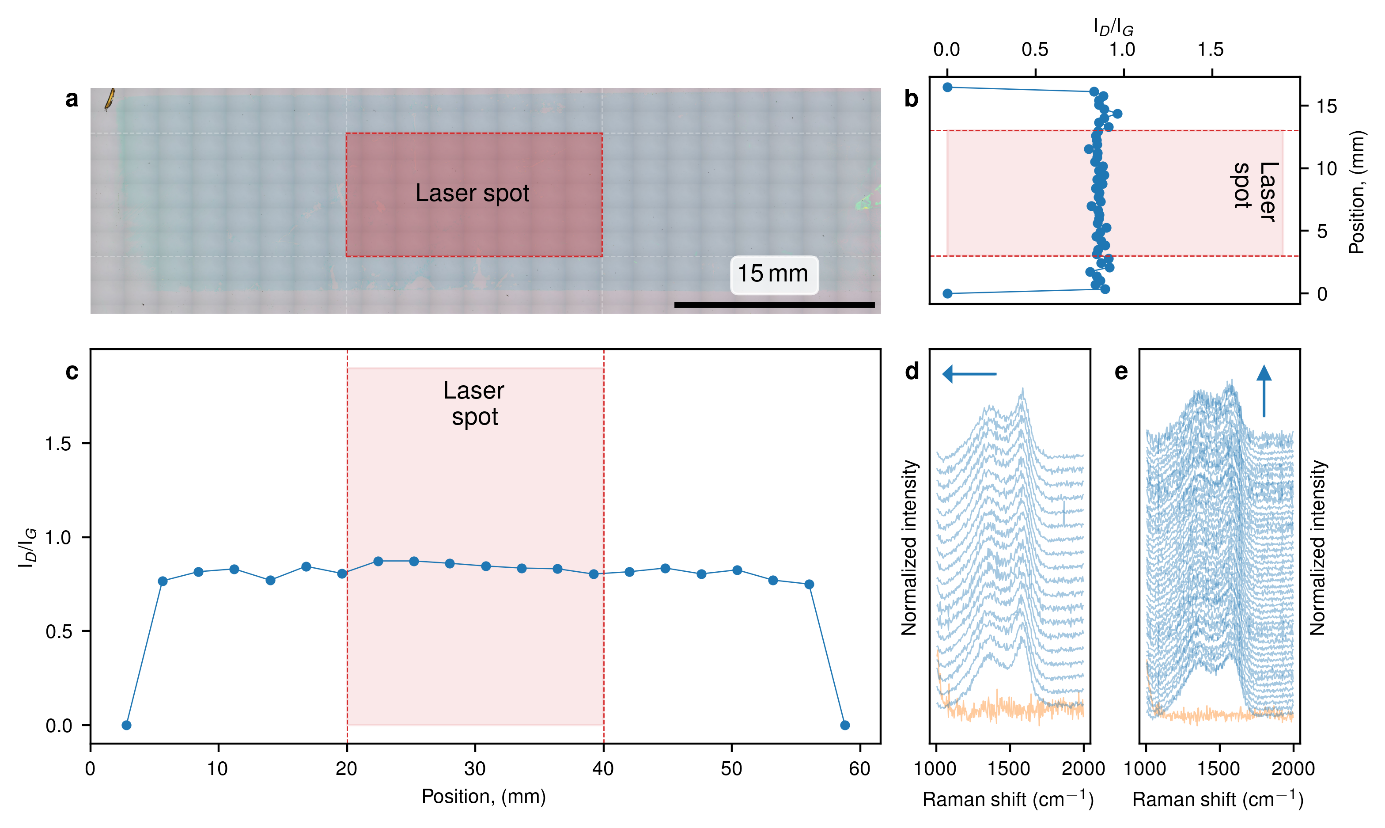
 **Figure S17.** Raman spectroscopy mapping of the disorder-degree uniformity across a 2 × 6 cm² MAC film grown using a 2 × 1 cm² laser spot. (a) Stitched optical micrographs (5× objective) of the transferred MAC film on SiO₂/Si. The red square marks the position of the laser spot during growth. (b) Spatial dependence of the I_D_/I_G_ ratio measured along the short axis of the sample. Spectra were collected along a line passing through the center of the laser-irradiated region. (c) Spatial dependence of the I_D_/I_G_ ratio measured along the long axis of the sample, with all spectra taken along a line through the center of the laser spot. (d, e) Representative individual Raman spectra corresponding to the data shown in panels c and b, respectively.


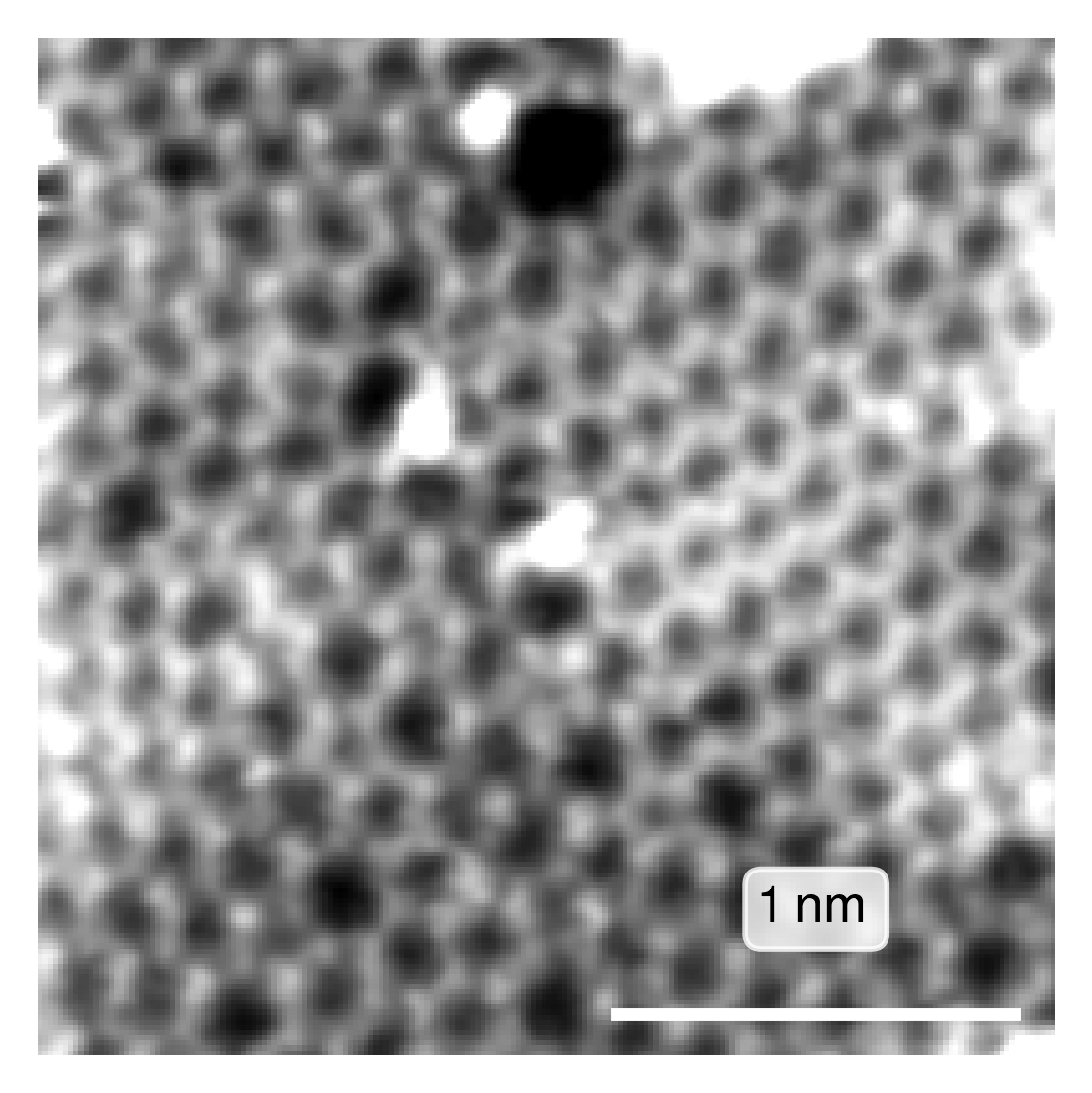


**Figure S18.** TEM image of MAC transferred from the non-irradiated region of the MAC/Cu sample.


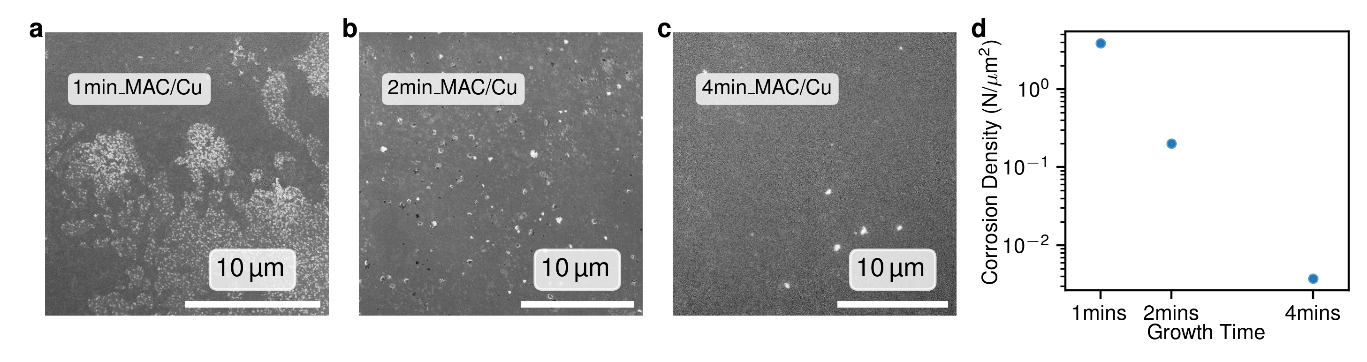


**Figure S19.** SEM images of MAC films grown for (a) 1 min, (b) 2 min, and (c) 4 min at constant temperature after undergoing corrosion tests. (d) Quantitative analysis of corrosion dot counts versus growth time.

Films grown for shorter durations (1 min and 2 min) exhibit numerous corrosion spots, indicative of incomplete or patchy coverage. In contrast, the 4 min sample showed a more uniform coating with significantly fewer corrosion sites, indicating that longer deposition times improve the MAC film’s coverage of the substrate. These results demonstrate that simply adjusting the growth duration effectively tunes the film continuity, thereby influencing its electrochemical and interfacial properties.


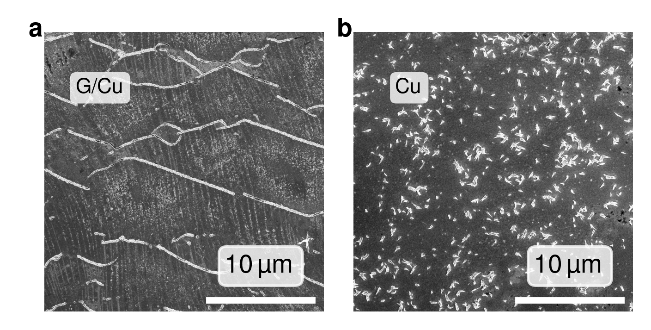


**Figure S20.** SEM images of (a) G/Cu and (b) bare Cu after undergoing corrosion tests.

Both G/Cu and Cu exhibit heavy corrosion. G/Cu tends to corrode primarily along grain boundaries, while the Cu shows more widespread, severe corrosion.


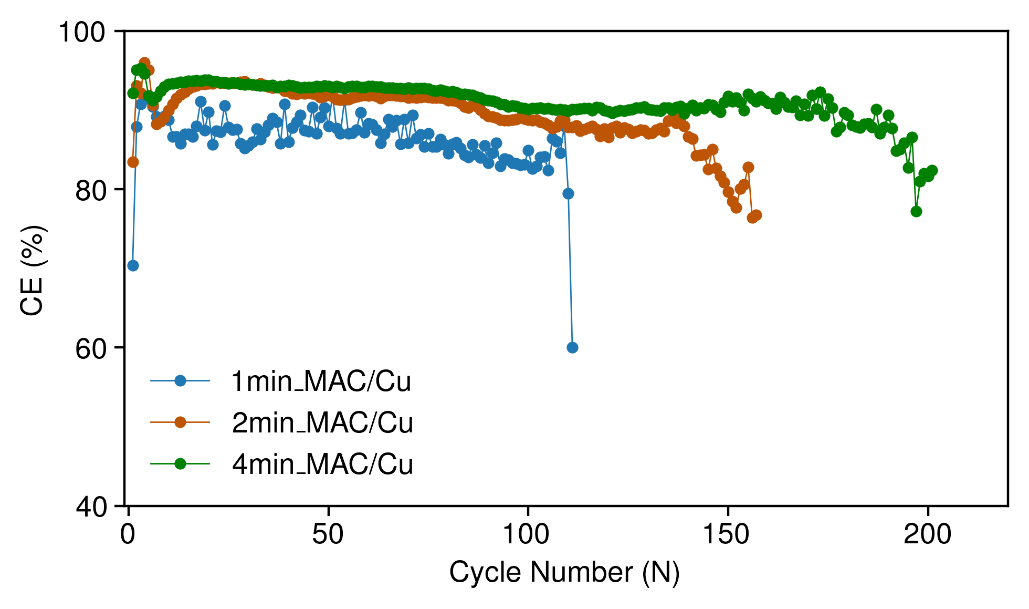


**Figure S21.** Influence of the growth time on cycling performance for MAC films.

Films grown for shorter durations (1 min and 2 min) exhibit limited CE retention, compared with the 4 min sample. This trend correlates with the improved film continuity observed in the corrosion tests: longer growth times lead to more complete and uniform MAC coverage, which mitigates localized failure and enhances electrochemical reversibility.


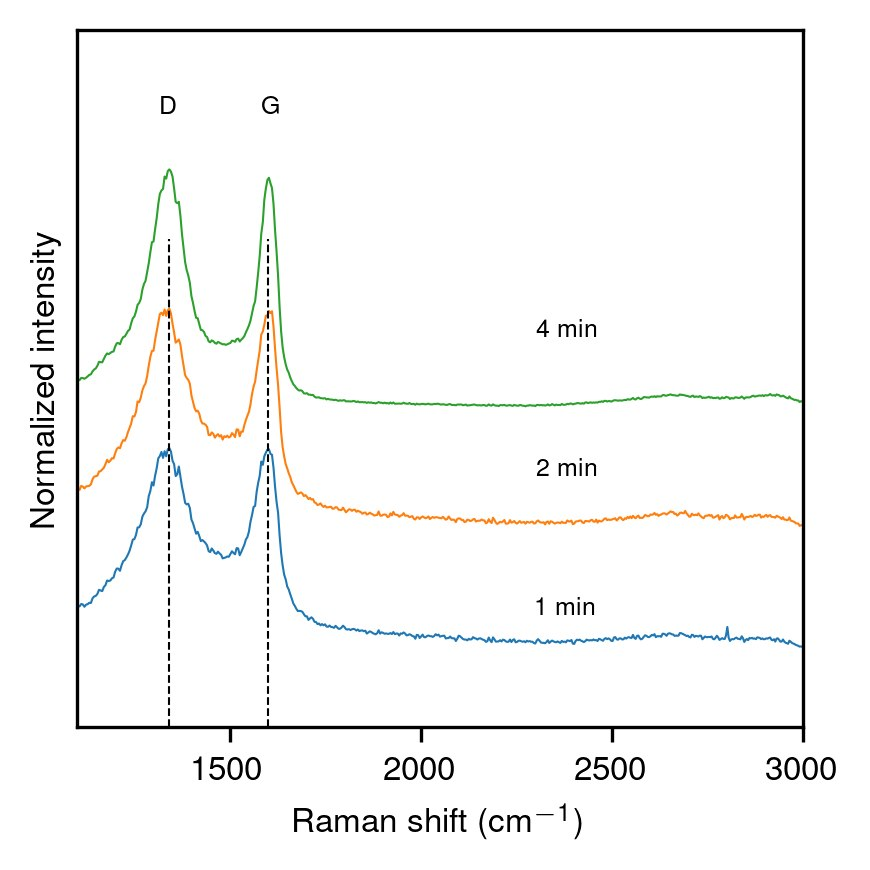


**Figure S22.** Raman spectra of MAC transferred onto SiO_2_ for different growth times.

The spectra for MAC grown at 1, 2, and 4 min all show similar I_D_/I_G_ ratios (0.82), indicating that the degree of disorder and the crystallite size (~1.1 nm) remain essentially unchanged with growth duration. This confirms that the level of structural disorder in MAC is primarily governed by growth temperature rather than deposition time. Therefore, variations in cycling stability with growth duration arise from differences in film continuity, not from changes in disorder or lithiophilicity.

**Table S1.** Electrochemical impedance of Li deposited after (0.5mA cm^-2^ for 1 hour) using three substrates. Values are obtained from fitting the Nyquist spectra with the circuit in Figure 5c and are reported before area normalization.

| Substrate | L_1_  [H] | R_ohm_  [Ω] | R_ct_  [Ω] | Q_2_  [F·s^(a-1)^] | a_2_ | Q_3_  [F·s^(a-1)^] | a_3_ |
| --- | --- | --- | --- | --- | --- | --- | --- |
| Cu | 6.68×10^-7^ | 1.04 | 19.26 | 2.87×10^-5^ | 0.81 | 1.39×10^-1^ | 0.33 |
| G/Cu | 2.59×10^-7^ | 0.56 | 28.93 | 1.68×10^-5^ | 0.84 | 8.57×10^-2^ | 0.22 |
| MAC/Cu | 2.77×10^-7^ | 0.61 | 14.25 | 2.31×10^-5^ | 0.86 | 1.08×10^-1^ | 0.26 |

Note SI:

The mechanical properties of MAC were characterized by using adhesion, fracture, and indentation measurements in separate publications. The adhesion energy between MAC and Cu, determined from 90° peeling and shear-force tests, is 85 J m⁻², about 13× higher than that of graphene on Cu.^[1]^ The fracture toughness, obtained from in situ crack propagation monitoring, gives a fracture energy of 45.7 J m⁻², roughly 3× higher than graphene.^[2]^ AFM nanoindentation on suspended MAC membranes yields a 2D elastic modulus (E₂D) centered at 115 N m⁻¹ and a breaking strength of 22 N m⁻¹, more than half that of single-crystal graphene.^[3]^

Note SII:

We derive the modified hard-soft acid-base (HSAB) expression assuming that the chemical potentials of the Li-atom and MAC can be written as

$$\mu_{Li}= \mu_{Li}^{0}+ \frac{1}{g_{Li}}q_{Li} + \sum\frac{n_{i}^{0}+n_{i}}{d_{i}}$$

$$\mu_{MAC, i} = \mu_{MAC}^{0}+\frac{1}{g_{i}}+ \frac{q_{Li}}{d_{i}}+\sum\frac{n_{j}}{d_{ij}}$$

where$\mu^{0}$ are the electronegativities, $g$are the local density of states at the Fermi level, $q_{Li}$ is the Lithium charge, $n^{0}, n$ are the initial charge inhomogeneity and induced charge on MAC, and $d_{i}, d_{i,j}$ are the distances between carbon atom and Li atom or between two carbon atoms, respectively. At equilibrium $\mu_{MAC, i}= \mu_{MAC}= \mu_{MAC}^{0}$, where the second equality follows from charge conservation $q_{Li}= -\sum n_{i}$, or equivalently from the assumption of a perfectly screened Lithium charge at large distances. We further note that a solution $\tilde{n_{i}}$ that fulfills the equilibrium condition for $q_{Li}= 1$, can be scaled to $n_{i}= \tilde{n_{i}}q_{Li}$ to obey equilibrium conditions for any $q_{Li}$. We hence write

$\mu_{Li} = \mu_{Li}^{0}+ \sum\frac{n_{i}^{0}}{d_{i}}+ \left( \frac{1}{g_{Li}}+ \sum\tilde{\frac{n_{i}}{d_{i}}} \right) q_{Li}$ (S1)

$\mu_{MAC}= \mu_{MAC}^{0}$ (S2)

where MAC as a surface with $\gg1$atoms has negligible hardness. Equations S1 and S2 now follow the classical HSAB formalism and setting $\mu_{Li}= \mu_{MAC}$ results in the Li adhesion energy

$$E = - \frac{\left( \mu_{Li}^{0}-\mu_{MAC}^{0}+ \sum\frac{n_{i}^{0}}{d_{i}} \right)^{2}}{2\left( \frac{1}{g_{Li}}+\sum\frac{\tilde{n_{i}}}{d_{i}} \right)}$$

We compute variations in energy using a first order Taylor expansion

$$dE = q_{Li}d\sum\frac{n_{i}^{0}}{d_{i}}+q_{Li}^{2}d\sum\frac{\tilde{n_{i}}}{d_{i}}$$

where we have used the relation

$q_{Li} = -\frac{\mu_{Li}^{0}-\mu_{MAC}^{0}+ \sum\frac{n_{i}^{0}}{d_{i}}}{\frac{1}{g_{Li}}+ \sum\tilde{\frac{n_{i}}{d_{i}}}}$

We achieve the highest agreement with DFT calculated results for a $q_{Li}$ = 1.05*e*, which is reasonably close to Mulliken charge of Li estimated to be 0.7*e* from DFT results.

Note SIII:

Applying HSAB for the interfacial energy calculations has a slight caveat, as the local density of states $g_{graphene}$ at the Fermi level is zero resulting in an infinitely large value of hardness and negligible Li adhesion energy. To obtain a non-trivial result, we take into account the whole energy dependence of the density of states $g\left( \epsilon\right)$. This results in

$$E = -\int_{\mu_{MAC}^{0}}^{\mu_{Li}^{0}} g\left( \epsilon\right) \left( \mu_{Li}^{0}-\epsilon\right)d\epsilon$$

assuming the hardness of bulk Li is infinitely small compared to the hardness of the 2D MAC surface.

**References**

[1] H. Zhang, A. K. Grebenko, K. V. Iakoubovskii, H. Zhang, R. Yamaletdinov, A. Makarova, A. Fedorov, R. SK, R. Shivajirao, Z. J. Tong, S. Grebenchuk, U. Karadeniz, L. Shi, D. V. Vyalikh, Y. He, A. Starkov, A. A. Alekseeva, C. C. Tee, C. M. Orofeo, J. Lin, K. Suenaga, M. Bosman, M. Koperski, B. Weber, K. S. Novoselov, O. V. Yazyev, C.-T. Toh, B. Özyilmaz, *Adv. Mater.* **2025**, 2419112.

[2] B. Shin, B. Ni, C.-T. Toh, D. Steinbach, Z. Yang, L. M. Sassi, Q. Ai, K. Niu, J. Lin, K. Suenaga, *Matter* **2025**, 8, 102000.

[3] C.-T. Toh, H. Zhang, J. Lin, A. S. Mayorov, Y.-P. Wang, C. M. Orofeo, D. B. Ferry, H. Andersen, N. Kakenov, Z. Guo, *Nature* **2020**, 577, 199.
